# Engineered Ultrasmall Nanoparticle Drug-Immune Conjugates with “Hit and Run” Tumor Delivery to Eradicate Gastric Cancer

**DOI:** 10.1002/adtp.202370009

**Published:** 2022-10-19

**Authors:** Li Zhang, Virginia Aragon-Sanabria, Anusha Aditya, Marcello Marelli, Tianye Cao, Feng Chen, Barney Yoo, Kai Ma, Li Zhuang, Thais Cailleau, Luke Masterson, Melik Z. Turker, Rachel Lee, Gabriel DeLeon, Sebastien Monette, Raffaele Colombo, Ronald J. Christie, Pat Zanzonico, Ulrich Wiesner, J. Anand Subramony, Michelle S. Bradbury

**Affiliations:** Department of Radiology, Sloan Kettering Institute for Cancer Research, New York, NY 10065, USA; MSK-Cornell Center for Translation of Cancer Nanomedicines, Sloan Kettering Institute for Cancer Research, New York, NY 10065, USA; Department of Radiology, Sloan Kettering Institute for Cancer Research, New York, NY 10065, USA; MSK-Cornell Center for Translation of Cancer Nanomedicines, Sloan Kettering Institute for Cancer Research, New York, NY 10065, USA; Department of Radiology, Sloan Kettering Institute for Cancer Research, New York, NY 10065, USA; MSK-Cornell Center for Translation of Cancer Nanomedicines, Sloan Kettering Institute for Cancer Research, New York, NY 10065, USA; AstraZeneca, One MedImmune Way, Gaithersburg, MD 20878, United States; Department of Radiology, Sloan Kettering Institute for Cancer Research, New York, NY 10065, USA; MSK-Cornell Center for Translation of Cancer Nanomedicines, Sloan Kettering Institute for Cancer Research, New York, NY 10065, USA; Department of Radiology, Sloan Kettering Institute for Cancer Research, New York, NY 10065, USA; MSK-Cornell Center for Translation of Cancer Nanomedicines, Sloan Kettering Institute for Cancer Research, New York, NY 10065, USA; MSK-Cornell Center for Translation of Cancer Nanomedicines, Sloan Kettering Institute for Cancer Research, New York, NY 10065, USA; Department of Chemistry, Hunter College, New York, NY 10065, USA; MSK-Cornell Center for Translation of Cancer Nanomedicines, Sloan Kettering Institute for Cancer Research, New York, NY 10065, USA; Department of Materials Science & Engineering, Cornell University, Ithaca, NY 14853, USA; AstraZeneca, One MedImmune Way, Gaithersburg, MD 20878, United States; AstraZeneca, Spirogen, QMB Innovation Centre, 42 New Road, London E1 2AX, UK; AstraZeneca, Spirogen, QMB Innovation Centre, 42 New Road, London E1 2AX, UK; MSK-Cornell Center for Translation of Cancer Nanomedicines, Sloan Kettering Institute for Cancer Research, New York, NY 10065, USA; Department of Materials Science & Engineering, Cornell University, Ithaca, NY 14853, USA; MSK-Cornell Center for Translation of Cancer Nanomedicines, Sloan Kettering Institute for Cancer Research, New York, NY 10065, USA; Department of Materials Science & Engineering, Cornell University, Ithaca, NY 14853, USA; Department of Radiology, Sloan Kettering Institute for Cancer Research, New York, NY 10065, USA; MSK-Cornell Center for Translation of Cancer Nanomedicines, Sloan Kettering Institute for Cancer Research, New York, NY 10065, USA; Laboratory of Comparative Pathology, Sloan Kettering Institute for Cancer Research, Weill Cornell Medicine, The Rockefeller University, New York, NY 10065, USA; AstraZeneca, One MedImmune Way, Gaithersburg, MD 20878, United States; AstraZeneca, One MedImmune Way, Gaithersburg, MD 20878, United States; MSK-Cornell Center for Translation of Cancer Nanomedicines, Sloan Kettering Institute for Cancer Research, New York, NY 10065, USA; Department of Medical Physics, Sloan Kettering Institute for Cancer Research, New York, NY 10065, USA; MSK-Cornell Center for Translation of Cancer Nanomedicines, Sloan Kettering Institute for Cancer Research, New York, NY 10065, USA; Department of Materials Science & Engineering, Cornell University, Ithaca, NY 14853, USA; Kavli Institute at Cornell for Nanoscale Science, Cornell University, Ithaca, NY 14853, USA; AstraZeneca, One MedImmune Way, Gaithersburg, MD 20878, United States; Department of Radiology, Sloan Kettering Institute for Cancer Research, New York, NY 10065, USA; MSK-Cornell Center for Translation of Cancer Nanomedicines, Sloan Kettering Institute for Cancer Research, New York, NY 10065, USA; Molecular Pharmacology Program, Sloan Kettering Institute for Cancer Research, New York, NY 10065, USA

**Keywords:** gastric cancer, HER2 targeted, nanoparticle drug-immune conjugate, silica, therapy, topoisomerase inhibitor, ultrasmall

## Abstract

Despite advances by recently approved antibody-drug conjugates in treating advanced gastric cancer patients, substantial limitations remain. Here, several key obstacles are overcome by developing a first-in-class ultrasmall (sub-8-nanometer (nm)) anti-human epidermal growth factor receptor 2 (HER2)-targeting drug-immune conjugate nanoparticle therapy. This multivalent fluorescent core–shell silica nanoparticle bears multiple anti-HER2 single-chain variable fragments (scFv), topoisomerase inhibitors, and deferoxamine moieties. Most surprisingly, drawing upon its favorable physicochemical, pharmacokinetic, clearance, and target-specific dual-modality imaging properties in a “hit and run” approach, this conjugate eradicated HER2-expressing gastric tumors without any evidence of tumor regrowth, while exhibiting a wide therapeutic index. Therapeutic response mechanisms are accompanied by the activation of functional markers, as well as pathway-specific inhibition. Results highlight the potential clinical utility of this molecularly engineered particle drug-immune conjugate and underscore the versatility of the base platform as a carrier for conjugating an array of other immune products and payloads.

## Graphical Abstract

A tunable first-in-class ultrasmall fluorescent core-shell silica nanoparticle bearing site-engineered anti-HER2 single chain variable fragments and topoisomerase inhibitors, eradicates HER2-expressing gastric tumors via a “hit and run” approach without tumor regrowth. Its favorable target-specific uptake and penetration, pharmacokinetic profile, and wide therapeutic index highlight its potential clinical utility for theranostic applications.

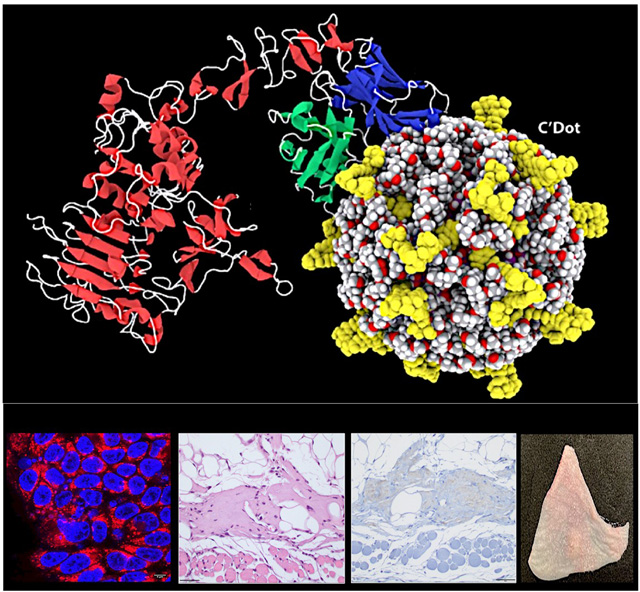

## 1. Introduction

Gastric cancer is one of the leading causes of cancer-related deaths. In 2020, over 1 million cases were estimated worldwide, representing nearly 6% of all cancer cases and ranking sixth among newly diagnosed cancers.^[1]^ While surgical management or minimally invasive procedures (i.e., endoscopy) remain primary treatment options for resection of localized^[2]^ or early-stage disease, respectively, the latter principally in Asia,^[2,3]^ most patients are diagnosed at advanced stages with poorer 5-year survival rates^[4]^ (i.e., ≈32% regional, 6% distant metastases) than those with more localized disease. Advanced or metastatic gastric cancer patients require pre- and/or post-operative chemotherapy, chemoradiation, and/or combinations of first and/or second-line systemic therapies, according to National Comprehensive Cancer Network (NCCN) guidelines,^[4,5]^ which improves survival and enhances the quality of life.^[6,7]^ Preferred treatment regimens are based on multiple factors, including cancer type/stage, biomarkers, such as human epithelial growth factor receptor 2 (HER2), and side effect profiles.

The development of advanced gastric cancer (AGC) has been linked to the overexpression of HER2 protein or amplification of the ERBB2 gene,^[8]^ which drives cell growth, survival, metastasis, and resistance.^[9]^ HER2 positivity rates are dependent on histologic subtype and tumor grade,^[10]^ with high rates, on average 20% (range: 12–23%),^[11,12]^ heralding a poor prognosis.^[13,14]^ Trastuzumab, a HER2 targeting monoclonal antibody (mAb) approved by the Food and Drug Administration (FDA) as an early first-line treatment,^[9,15]^ has shown initial clinical benefits in HER2 overexpressing AGC when added to first-line chemotherapies (i.e., fluoropyrimidine plus cisplatin) as compared with chemotherapy alone.^[16]^ A key limitation of this agent is the eventual development of resistance.^[15]^ More recently, a combination of pembrolizumab, trastuzumab, and chemotherapy was approved as a new first-line standard of care regimen for patients harboring untreated and unresectable or metastatic HER2 positive (HER2+) AGC (Phase III KEYNOTE-811^[17–19]^). For those patients who fail prior treatments, the antibody-drug conjugate (ADC), fam-trastuzumab deruxtecan-nxki (DS-8201a), bearing a topoisomerase I inhibitor^[20]^ has been used as a second line (or beyond) agent based on results of the DESTINYGastric01 Phase II trial.^[17,19]^ Table S1, Supporting Information, highlights several FDA-approved products for AGC, with pivotal randomized trials of representative HER2 targeting therapies summarized in Table S2, Supporting Information.^[21]^ Other selected HER2-directed therapies,^[22]^ including trastuzumab emtansine (T-DM1), pertuzumab, and lapatinib, have not shown clinical benefit in HER2+ AGC patients despite their success in treating other tumor types.^[23]^

ADCs (Tables S1 and S2, Supporting Information), have demonstrated rapid growth in recent decades for treating a variety of cancers, with 5 ADCs alone approved over the past 2 years. Despite these successes, several limitations have persisted,^[24]^ including off-target toxicity, drug resistance, relatively low drug-antibody ratios, and poor solid tumor penetration,^[25,26]^ that, in turn, may reduce efficacy.^[25]^ As a result, several ADCs used in the clinic have been discontinued. Such developments have spurred explosive growth over the past decade in a diverse array of alternative targeted nanodelivery vehicles used as monotherapies or as part of combinatorial regimens^[27]^ for treating AGC; these have been summarized in a number of excellent comprehensive reviews.^[9,28,29]^ Engineered inorganic particle therapies, for instance, have been developed and conjugated with immune components^[30–32]^ for treating HER2+ gastric cancers. While therapeutically effective, none of these have led to a complete tumor regression. Other classes of therapeutic nanoprobes,^[33–35]^ including polymeric systems^[36,37]^ and lipid-based carriers,^[38,39]^ have also been investigated for treating a number of different gastric cancer models (i.e., HER2− to study resistance), utilizing one or more small molecule drugs, and/or targeting alternative tumor antigens.^[40,41]^ While these foregoing approaches for improving the diagnosis and treatment of AGC are promising, the multifunctional platforms are all relatively large in size (>20 nm), which can limit their efficient penetration, diffusion, and distribution within solid tumors. Additional challenges include off-target toxicity and protein corona formation,^[42]^ among others, which can affect pharmacokinetic (PK) profiles and therapeutic responses.^[24]^

Importantly, despite the growing number of nanoparticle drug-immune conjugates being evaluated preclinically, only a vanishingly small proportion of these are advanced to the clinic. Notable among them are ultrasmall (sub 8-nm diameter) and ultrabright fluorescent core–shell silica nanoparticles, Cornell dots (C dots), comprised of a (Cy5) dye-encapsulating silica core and short poly(ethylene glycol) (PEG) ligand shell, originally synthesized via a modified Stöber process in alcohol.^[43–45]^ Second-generation C dots synthesized in water,^[46]^ referred to as Cornell prime dots (C′ dots), enable improved control over structural and compositional particle characteristics translating into substantially improved biological properties. Size control of the silica core down to the single atomic layer^[46]^ with diameters maintained below the cut-off for renal clearance and narrow particle size distributions (±20% for core sizes down to 30Å),^[47]^ assures that all particles, not just a fraction, are renally cleared. Dense (≈1 nm thick) PEG brushes maximize steric stability via robust and mechanistically well-understood PEGylation protocols,^[48]^ in turn, minimizing protein corona formation and enabling high drug loading capacities via insertion of hydrophobic drugs into the PEG brush (vide infra). Further, quantitative elucidation of and control over heterogeneities in C′ dot surface chemical properties using high-performance liquid chromatography (HPLC) for C′ dot quality control (QC),^[49,50]^ enhances an array of specific C′ dot-induced therapeutic responses. Finally, multifunctional surface modification via post-PEGylation surface modification by insertion (PPSMI) procedures decouples particle stabilization via PEGylation from further silica surface coupling reactions.^[51]^ In turn, this leads to well-controlled functional ligand numbers per particle that strongly correlate with key biological properties including targeting affinity and therapeutic efficacy (vide infra). Designed as a multivalent theranostic platform, we have previously conjugated the C′ dot surface with a variety of targeting ligands,^[51–53]^ small-molecule drugs,^[54–56]^ and radiolabels^[57]^ for image-guided surgical^[58,59]^ and therapeutic^[54,55]^ applications, each time carefully optimizing ligand numbers and particle architecture in order to maximize desired biological properties. Drug-immune conjugate C′ dots developed here have not been described before, however.

Part of the success of ADCs lies in the high targeting efficiency of antibodies (Abs), whose multivalent architecture leads to high target specificity and affinity. It is, therefore, attractive to contemplate how to harness these desirable Ab properties for a renally clearable molecularly engineered nanodelivery vehicle that can simultaneously overcome some limitations of ADCs. In the C′ dot studies noted above, targeting ligands were based on small molecules (e.g., prostate-specific membrane antigen [PSMA] inhibitor,^[53]^ folic acid^[56]^) or peptides (e.g., cyclic arginine-glycine-aspartic acid, cRGD^[51]^ or alpha-melanocyte-stimulating hormone, *α*MSH),^[52]^ which only marginally added to particle size, even at relatively high ligand numbers (i.e., 15–20), thereby maintaining the desirable bulk renal clearance properties of C dots.^[60,61]^ Compared to C′ dots, Abs are relatively large (≈15 nm). In order to combine the favorable particle biodistribution profiles with the high target specificity and affinity of Abs, Ab fragments are a promising alternative.^[62]^ To this end, anti-HER2 single-chain fragments were recently conjugated to dual-modality (PET-optical) C′ dots for imaging HER2 overexpressing breast cancer,^[63]^ leading to a sub-8-nm target-or-clear tracer with high tumor-targeting efficiency, target-to-background ratios, and bulk renal clearance. This particle immunoconjugate, however, was not designed for targeted therapy.

Herein, we report on the rational design and translational development of a first-in-kind ultrasmall nanoparticle drug-immune conjugate, anti-HER2 scFv-SG4015-PEG-Cy5-C′ dots, for dual-modality imaging and therapy of HER2+ expressing gastric cancer utilizing a “hit and run” delivery approach. Designed for therapeutic agents, this approach reflects the evolution of the “target-or-clear” paradigm descriptive of our diagnostic imaging agents.^[57]^ It signifies a process by which the particle-based drug-immune conjugate hits its target and delivers a cytotoxic payload, while conjugates not reaching the tumor are efficiently cleared reducing off-target effects. To that end, we built on the considerable efforts that have been devoted to engineering and advancing multiple surface chemical components and particle conjugation strategies for expanding platform functionality in a reproducible manner, while maintaining sub-8 nm particle sizes and achieving favorable biological performance (vide supra). Using an efficient click chemistry bioconjugation procedure, 6–7 nm sized Cy5 dye-encapsulating and PEGylated C′ dots (i.e., PEG-Cy5-C′ dots) were functionalized with site-engineered anti-HER2 single chain variable fragments (scFv), as reported previously, followed by the conjugation of efficacious topoisomerase inhibitors, SG4015, via enzymatically cleavable linkers, and deferoxamine (DFO) moieties for radiolabeling. The numbers of anti-HER2 scFv ligands and drugs were optimized on a per-particle basis. A dose escalation strategy was designed to assess suitable particle dosing strategies based on the median lethal dose (LD_50_); the median effective dose (ED_50_) and therapeutic index (TI) were also determined. This multifunctional and renally-clearable platform design met designated success criteria and addressed key translational challenges associated with particle-based drug-immune conjugate developments in a non-metastatic NCI-N87 xenograft model, including: 1) tumor eradication and survival benefit over controls without evidence of tumor regrowth or dose-limiting toxicity; 2) high target-specific accumulations and target-to-background ratios difficult to achieve with larger-size (>10 nm) platforms; 3) favorable whole-body PK; and 4) wide therapeutic index. Finally, mechanistically we showed that the conjugate inhibited downstream signaling intermediates driving oncogenesis.

## 2. Results and Discussion

### 2.1. Site-Specific Engineering of Anti-HER2 scFv Fragments

**Figure 1.**
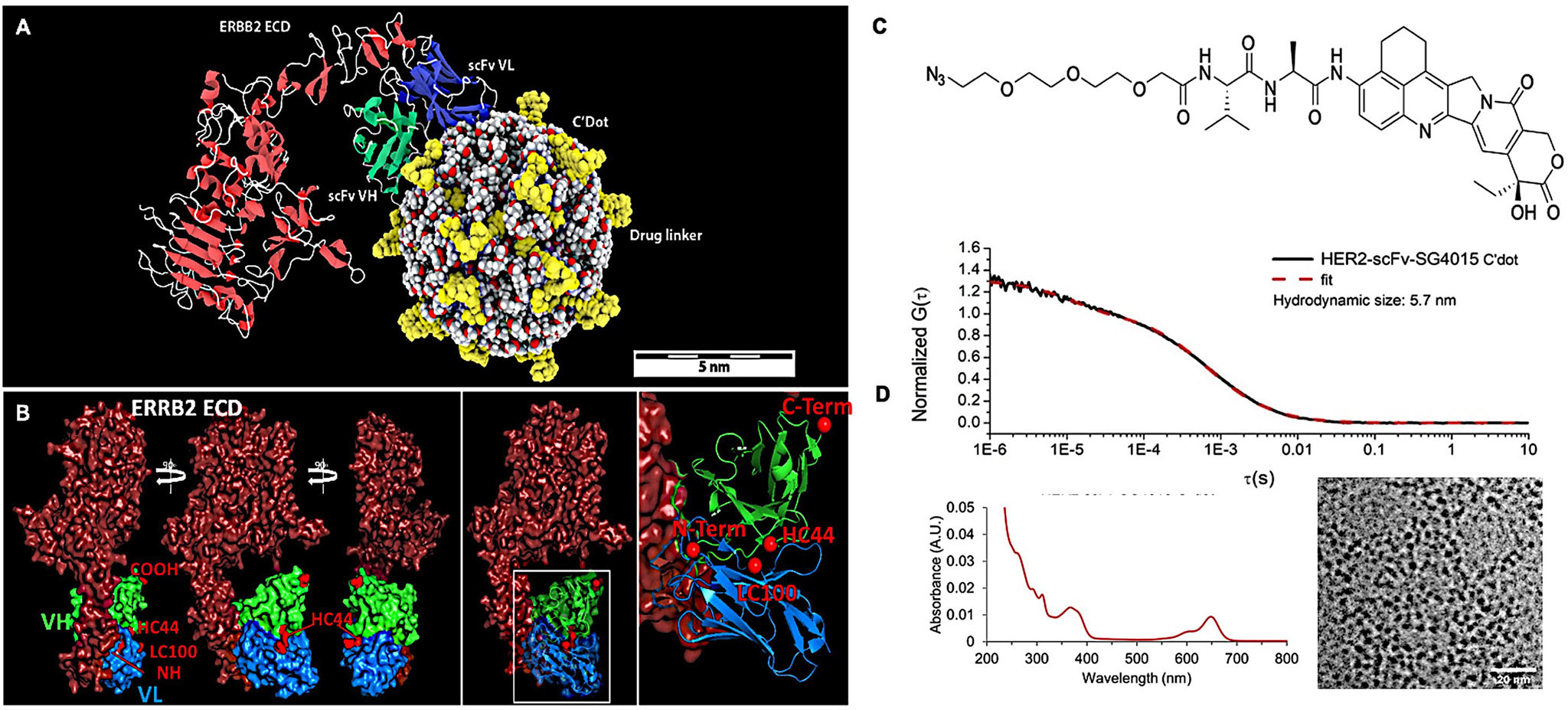
DFO-scFv-SG4015-PEG-Cy5-C′ dot conjugate structure and characterization. A) 3D rendering of complete target/immune-drug particle complex with ERBB2 extracellular domain (ECD, red) and scFvs (VL and VH regions blue and green, respectively) with PEG-Cy5-C′ dot (white/red space-filled) and SG4015 with linker (yellow). B) Rotated space-filling model depicting contact sites between the ERBB2 ECD (maroon) and scFvs directed to ERBB2 ECD with four sites of nnAA integration (red; N-Term, LC100, HC44, and C-Term, inset). Sites were rationally designed to be distal to the antigen-binding domains and surface-exposed to enable efficient conjugation. C) Structure of topoisomerases inhibitor (SG4015) payload. D) Conjugate characterization: (top) FCS correlation curve with fit; (bottom, left) UV–vis spectrum showing clear fingerprints of DFO (≈250 nm), DBCO (double peak ≈300 nm), drug (≈350–400 nm), and Cy5 dye (Abs. max ≈650 nm); (bottom, right) TEM of the final particle (scale bar 20 nm).

A single-chain antibody fragment (scFv) was used as a targeting ligand due to its small size (25 kDa) and retained specificity for its antigen (Figure 1A). For this study, an scFv targeting the Her2/neu receptor, based on the antibody 4D5, was constructed using a variable light/heavy chain (VL-linker-VH) orientation, as reported previously and shown in Figure 1B and Figure S1, Supporting Information.^[63]^ Site-specific conjugation of the scFv fragment was conducted to control the site and number of conjugations; this ensured that the scFv contained a single conjugation site remote from its antigen-binding function (Figure S1, Supporting Information). The position of the conjugation site was controlled by genetically encoding a non-natural amino acid in engineered cells expressing the pyrrolysine tRNA synthetase/tRNApyl pair from *Methanosarcina mazei*, as previously described.^[63–65]^

We introduced a lysine analog containing an azide moiety (N_6_-((2-azidoethoxy) carbonyl)-L-lysine; AzK) that enables azide-alkyne cycloaddition or click chemistry. This conjugation chemistry is bioorthogonal, efficient, and results in the formation of a highly stable heterocyclic triazole. Surface-exposed residues consisting of two heavy chain (HC) positions (i.e., C-terminal and HC44, an intragenic site position) and two light chain (LC) sites (i.e., N-terminal and LC100; Figure 1B and Figure S1, Supporting Information) were selected on the basis of available crystal structures used to model the scFv fragment,^[66]^ as well as on their ability to enable efficient conjugation while retaining antigen-binding function. An anti-HER2 scFv containing AzK at position HC44 was selected for subsequent C′ dot bioconjugation studies based on stability, efficient conjugation, and antigen-binding properties (Figure 1 and Figure S1, Supporting Information).

### 2.2. Synthesis of SG4015 Conjugated to Enzymatically Cleavable Linkers

The structure of the cytotoxin SG4015 is shown in Figure 1C and Figure S2A, Supporting Information, the latter indicating atom assignments for ^1^H NMR analysis, along with its ^1^H NMR spectrum (Figure S2B, Supporting Information). SG4015 is an efficacious topoisomerase inhibitor, a derivative of the camptothecin class, and was prepared to contain an azide-Val-Ala cleavable dipeptide linker for payload attachment to dibenzocyclooctyne (DBCO) functional groups available on the functionalized C′ dot surface (see below). The linker is cleaved by endosomal proteases, such as cathepsin B, as described in an earlier publication,^[67]^ thereby releasing the active topoisomerase I inhibitor.

### 2.3. Synthesis and Characterization of (Ctr/)scFv-SG4015-PEG-Cy5-C′ Dots with and without DFO-Conjugation

Multifunctional scFv-SG4015-PEG-Cy5-C′ dots and Ctr/scFv-SG4015-PEG-Cy5-C′ dots (against human metapneumovirus58; see Experimental Section), with and without a DFO chelator, were synthesized as part of a multi-step process (Figure S3, Supporting Information) that enables integration of up to six different functional moieties into a single 6–7 nm fluorescent core–shell silica nanoparticle: encapsulated Cy5 fluorescent dye, PEG stealth layer, DBCO, DFO chelator, SG4015 cleavable drug linker conjugate, and single-chain variable heavy (*V*_H_) and light (*V*_L_) chain Ab fragments (scFv). Aminated and PEGylated dots (NH_2_-PEG-Cy5-C′ dots or simply NH_2_-C′ dots) were prepared using previously published protocols (Figure S3, Supporting Information) and were characterized using gel permeation chromatography (GPC), fluorescence correlation spectroscopy (FCS), and UV–vis spectroscopy (data not shown).^[63]^

To synthesize DFO-scFv-SG4015-PEG-Cy5-C′ dots, aminated NH_2_-PEG-Cy5-C′ dots were first reacted with p-SCN-Bn-DFO (DFO-NCS) to form DFO-PEG-Cy5-C′ dots. DBCO-PEG_4_-N-hydroxysuccinimidyl ester (DBCO-PEG_4_-NHS ester) was then added (pH 7.4), yielding DFO-DBCO-PEG-Cy5-C′ dots with controllable DBCO surface density. Separation of unreacted DFO and DBCO moieties was achieved by passing the reaction mixture through a GPC column. Subsequently, DFO-DBCO-PEG-Cy5-C′ dots were mixed with pre-synthesized anti-HER2 scFv-azide (position HC44, Figure S3, Supporting Information) in phosphate-buffered saline (PBS) and reacted overnight, followed by the addition of azide-SG4015 drug linker, to yield DFO-scFv-SG4015-PEG-Cy5-C′ dots. The surface density of components, namely DFO, DBCO, scFv, and SG4015, were carefully optimized to ensure a final particle composition and size that facilitated bulk renal clearance while maintaining low reticuloendothelial system (RES) uptake. To synthesize scFv-SG4015-PEG-Cy5-C′ dots without DFO chelators, the DFO conjugation step was excluded from the above procedure.

As-synthesized scFv-SG4015-PEG-Cy5-C′ dots were evaluated using a combination of techniques, including GPC, FCS, transmission electron microscopy (TEM), and UV–vis spectroscopy (Figure 1).^[43,46,51,63]^ No impurities were identified by GPC utilizing a previously established particle purification scheme (Figure S3, Supporting Information) showing the successful removal of all unreacted reagents from the reaction solution. The average HD of GPC-purified DFO-scFv-SG4015-PEG-Cy5-C′ dots was determined to be 5.7 nm by fitting the FCS correlation curve, consistent with TEM observations (Figure 1D).

In-depth C′ dot immune-drug conjugate characterization was performed to accurately assess the attachment of SG4015-azide to the particle surface (Figure S4, Supporting Information). Acquired UV–vis absorbance spectra of scFv-SG4015-PEG-Cy5-C′ dots (drug-per-particle ratio, or DPR of ≈40) show well-distinguished absorption peaks at ≈360 nm (SG4015) and 651 nm (C′ dots), respectively (Figure S4A, Supporting Information); a similar DPR was found for DFO-functionalized particles. There was no significant change in the appearance of these absorption peaks over a 19-day period post-synthesis (Figure S4A, Supporting Information), nor in the corresponding HPLC chromatograms acquired with ≈360 nm detection (Figure S4B, Supporting Information). To quantify the number of functional ligands per C′ dot, as shown in detail elsewhere, the UV–vis absorbance was fit by a linear combination of the absorption spectra of each of the individual components including Cy5, DBCO, DFO, and scFv.^[51,63]^ As a result, the UV–vis absorbance of scFv-SG4015-PEG-Cy5-C′ dots was successfully deconvoluted, and numbers of Cy5, DBCO, scFv, and SG4015 moieties per C′ dot were estimated to be around 1.9, 22, 1.4, and 40, respectively. Next, we evaluated the payload (i.e., drug-linker) stability of scFv-SG4015-PEG-Cy5-C′ dots while stored in PBS (4 °C, pH 7.4) or during incubation with serum (mouse and human). As shown in Figure S4C, Supporting Information, quantitative analyses based on UV–vis absorbance spectra showed <3% pre-release of the drug from scFv-SG4015-PEG-Cy5-C′ dots (DPR 40) after storing in PBS for 19 days. Slightly higher drug release (i.e., 5–7%) was observed upon incubation of the same construct with human or mouse serum for at least 140 h (Figure S4D, Supporting Information). Based on these results, a DPR ≈40 and about 1.4 scFv per C′ dot were used for in vitro HER2 active targeting studies, radiolabeling, and in vivo HER2 targeted PET imaging, dose escalation, and therapeutic studies.

### 2.4. In Vitro Analysis of scFv-SG4015-C′ Dot Specificity, Binding Affinity, Potency, and Internalization

**Figure 2.**
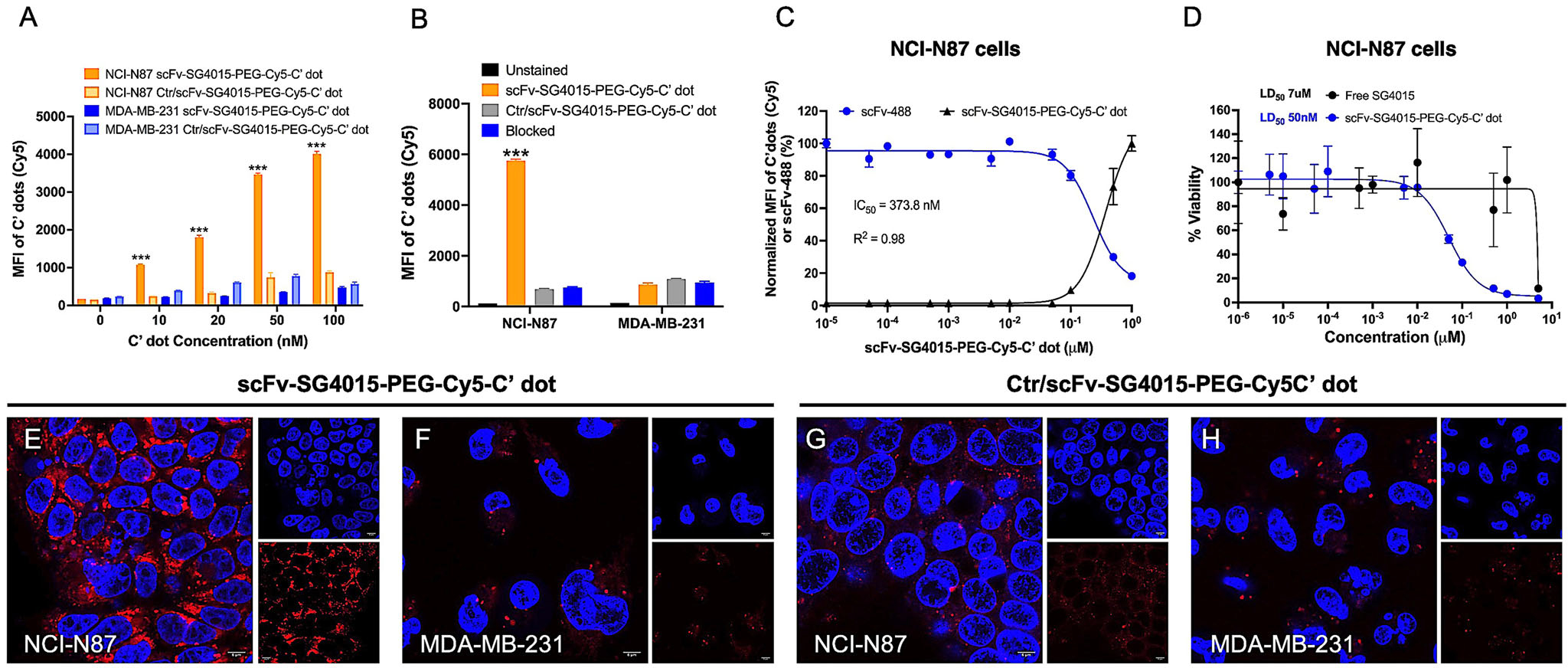
Target-specific uptake, half-maximal concentration (IC50), and median lethal dose (LD50) of scFv-SG4015-PEG-Cy5-C′ dots. A) In vitro targeted uptake of scFv- (and Ctr/scFv)-SG4015-PEG-Cy5-C′ dots in HER2+ NCI-N87 versus HER2− MDA-MB-231 cell lines as a function of particle concentration (***, *p* < 0.001). B) HER2 targeting specificity of scFv (and Ctr/scFv)-SG4015-PEG-Cy5-C′ dots (100 nM). For blocking assays, a 20-fold excess of the free scFv-azide fragment (2 μM) was used. (***, *p* < 0.001). C) Concentration-dependent inhibition of scFv-488 binding to NCI-N87 cells by scFv-SG4015-PEG-Cy5-C′ dots. Each data point represents the mean ± s.d. of three replicates. D) LD50 curves for NCI-N87 cells treated with free SG4015 (LD_50_ = 7 μM), or scFv-SG4015-PEG-Cy5-C′ dots (LD_50_ = 50 nM). E–H) Confocal microscopy of scFv-(and Ctr/scFv)-SG4015-PEG-Cy5-C′ dots (100 nM) internalization in NCI-N87 cells (E,G) and MDA-MB-231 cells (F,H). Blue and red colors represent DAPI and Cy5, respectively. Insets show corresponding DAPI (upper) and Cy5 (lower) channels. Scale bars: 6 μm.

In vitro studies were conducted with non-DFO-functionalized C′ dots, as these did not require radioisotopic labeling. To demonstrate HER2-specific targeting capabilities, scFv-SG4015-PEG-Cy5-C′ dots and Ctrl/scFv-SG4015-PEG-Cy5-C′ dots (control) were incubated with two well-established cancer cell lines exhibiting distinctly different HER2 expression signatures: NCI-N87 (HER2+, ≈3.2 × 10^6^ receptors per cell), a human gastric cancer cell line, and MDA-MB-231 (HER2−, 7 × 10^4^ receptors per cell),^[68]^ a human triple-negative breast cancer line reported on previously.^[63]^ Importantly, NCI-N87 cells exposed to GPC-purified scFv-SG4015-C′ dots, about ≈1.4, scFvs per particle^[63]^ showed a significant concentration-dependent increase in the Cy5 median fluorescence intensity (MFI) by flow cytometry; specifically, a fourfold MFI enhancement was observed for concentrations ranging from 10 to 100 nanomolar (nM over a 4-h particle exposure time at 37 °C (Figure 2A).

Further, MFI values for NCI-N87 cells were, on average, about eightfold higher than those for MDA-MB-231 cells (i.e., 4000 vs ≈500 arbitrary units (a.u.)) at a 100 nM particle concentration (Figure 2A), consistent with a target-specific uptake mechanism in HER2+ cells. By contrast, incubation of NCI-N87 cells with Ctr/scFv-SG4015-PEG-Cy5-C′ dots (control) over this same concentration range showed only about a twofold MFI enhancement, with a fivefold reduction in cellular uptake at 100 nM relative to the targeted particle probe (Figure 2A). As expected, negligible uptake of Ctr/scFv-SG4015-PEG-Cy5-C′ dots in MDA-MB-231 cells was observed. Receptor-blocking studies confirmed high HER2 target specificity of scFv-SG4015-PEG-Cy5-C′ dots (Figure 2B). A ≈90% reduction in the targeted uptake of 100 nM scFv-SG4015-PEG-Cy5-C′ dots was found in NCI-N87 cells after pre-incubation with a 20-fold excess of the free scFv-azide fragment (Figure 2B), but not with cells exposed to Ctr/scFv-SG4015-PEG-Cy5-C′ dots (100 nM). No significant difference in MDA-MB-231 cell binding was found among the three groups (Figure 2B).

In addition, the relative HER2 receptor binding affinity of scFv-SG4015-PEG-Cy5-C′ dots was assessed in terms of the IC_50_ value using NCI-N87 cells, scFv-488 fragments (40 nM), and fluorescence activity single-cell sorting analysis (FACS), according to previously established methods.^[63]^ An IC_50_ value of 373.8 (Figure 2C) was found for scFv-SG4015-PEG-Cy5-C′ dots, which was fairly equivalent to that found previously for the diagnostic platform, scFv-PEG-Cy5-C′ dots (i.e., IC_50_ = 305.7 nM), but slightly larger than that for the free scFv-azide fragment (IC_50_ = 107.5 nM).^[63]^ Although we can only speculate about the reasons for this observed sequence of IC_50_ values here, it likely is associated with an increase in steric hindrance as one moves from the free scFv to a particle-conjugated scFv, and then to both fragment and drug-conjugated particles.

The cytotoxicity of scFv-SG4015-PEG-Cy5-C′ dots (DPR ≈40) versus free drug SG4015 was evaluated in HER2+ NCI-N87 cells exposed to a range of concentrations (10^−6^ to 1 μM) for 7 days. Viability was assessed as a percentage of the respective untreated controls and used to calculate a median lethal dose (LD_50_) of 50 nM in NCI-N87 cells (Figure 2D). Confocal microscopy demonstrated higher target-specific delivery and internalization of scFv-SG4015-PEG-Cy5-C′ dots (100 nM) by NCI-N87 cells (Figure 2E), as compared with biological (i.e., HER2− MDA-MB-231 cells, Figure 2F) or particle (i.e., Ctr/scFv-SG4015-PEG-Cy5-C′ dots) controls (Figure 2G,H). These results underscore the need to achieve precise surface chemical control of a multi-step process, accompanied by rigorous product characterization, in order to create highly potent and target-specific particle drug-immune conjugates with favorable biological and toxicological responses.

### 2.5. Endpoint Criteria for In Vivo Molecular Imaging and Therapeutic Studies in the NCI-N87 Xenograft Model

The translational potential of this first-in-class C′ dot drug-immune conjugate was assessed relative to controls using an NCI-N87 xenograft model given the complexities and challenges associated with the generation and monitoring of orthotopic AGC models, for example, tumor cell shedding into the peritoneal cavity, limited tissue penetration with conventional optical approaches, need for specialized endoscopy tools, among others.^[69,70]^ Multiple endpoint criteria were specified to capture the following target and off-target biological responses of the C′ dot drug-immune conjugate: i) high tumor activity-concentrations, ii) target-specific tissue penetration and distribution, iii) therapeutic index indicating a wide safety margin, and iv) high treatment efficacy. For the first endpoint, specific tumor accumulations of 10–15 percentage injected dose per gram of tissue (%ID/g) were prescribed for the ^89^Zr-labeled scFv-SG4015-PEG-Cy5-C′ dot; these were expected to be significantly greater than those for the radiolabeled particle control, accompanied by significantly higher tumor-to-background ratios (or enhancements). The 10–15% activity concentration is several-fold higher than that expected for a uniformly distributed agent, or 4% ID/g, the latter estimated to be 100% of the injected dose (ID) distributed throughout a ≈25-gram murine body mass. Regarding the second endpoint, the targeted particle therapeutic is expected to penetrate, accumulate, and distribute within tumor tissue specimens. For the third endpoint, the therapeutic index should exhibit a greater than twofold difference between the median lethal dose (LD_50_) and median effective dose (ED_50_), as defined by the FDA Code of Federal Regulations (CFR).^[71]^ Finally, statistically significant (*p* < 0.05) increases in tumor regression and survival benefit should be observed for the targeted particle therapeutic using optimized particle doses, as against the controls, without associated dose-limiting toxicity.

### 2.6. Pharmacokinetic Profiles, Renal Clearance, and Target-Specific Uptake of ^89^Zr-DFO-scFv-SG4015-PEG-Cy5-C′ Dots

**Figure 3.**
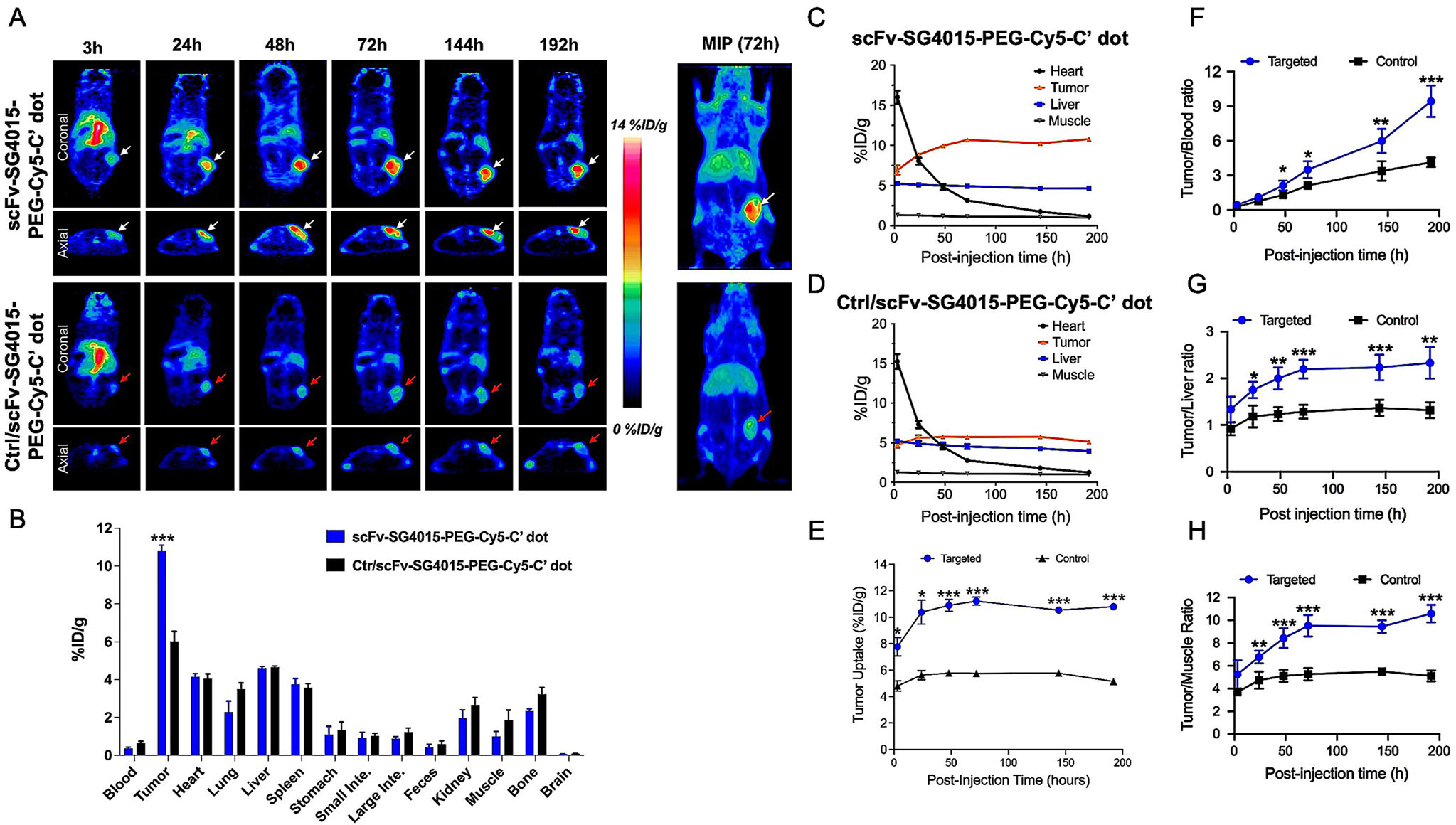
In vivo HER2 targeted uptake, penetration, and retention in NCI-N87 xenografted mice on PET imaging and biodistribution studies. A) Longitudinal analysis of coronal and axial tomographic PET images acquired at 3-, 24-, 48-, 72-, 144- and 192-h post i.v. injection of radiolabeled particle immunoconjugates in NCI-N87 tumor-bearing mice (*n* = 6 for each group) as follows—targeted group: ^89^Zr-DFO-scFv-SG4015-PEG-Cy5-C′ dots (top panel) and isotype control group: ^89^Zr-DFO-Ctr/scFv-SG4015-PEG-Cy5-C′ dots (bottom panel). For each group, maximum intensity projection (MIP) images were also acquired at 72 h p.i. (right panels). Tumors were marked with white and red arrows for the targeted and control groups, respectively. B) Biodistribution profiles for both groups at 192 h p.i. ***p < 0.0001. The two degrees-of-freedom F-test was followed by pairwise *t*-tests that were adjusted for multiple comparisons using the Holm method. Each data point represents the mean ± s.e.m of six replicates. C,D) Time-activity curves showing major organ uptake of ^89^Zr-labeled nanoparticles in NCI-N87 tumor bearing-mice injected with a targeted: ^89^Zr-DFO-scFv-SG4015-PEG-Cy5-C′ dots (C) or isotype control: ^89^Zr-Ctr/scFv-SG4015-PEG-Cy5-C′ dots (D). E) Comparison of tumor uptake between groups. F–H) Comparison of tumor-to-blood (F), tumor-to-liver (G), and tumor-to-muscle (H) ratios for both groups. *n* = 6 animals per group, ***p < 0.001, **p < 0.01, *p < 0.05.

To assess target-specific uptake, bulk renal clearance, and PK profiles in NCI-N87 tumor-bearing mice, 5.7 nM DFO-scFv-SG4015-PEG-Cy5-C′ dots (or Ctr/scFv-SG4015-PEG-Cy5-C′ dots), with a DPR of ≈40, were radiolabeled with ^89^Zr (*t*_1/2_ = 78.4 h). Radiochemical purity was estimated to be greater than 99% using radio-TLC (Figure S5, Supporting Information) after initial purification with a PD-10 column. Radiochemical yields were greater than 60%, and specific activities ranged from 3300 to 5700 Ci mmol^−1^ for ^89^Zr-labeled targeted and control particle tracers using a starting dose of 1 nanomole. Serial PET imaging was conducted in HER2+ NCI-N87 tumor-bearing mice (Figure 3A, upper panel; Figure S6 and Table S3, Supporting Information) at 3-, 24-, 48-, 72-, 144-, and 192-h after intravenous injection (i.v.) of 200–300 mCi ^89^Zr-DFO-scFv-(or Ctr/scFv)-SG4015-PEG-Cy5-C′ dots (*n* = 3 mice per cohort; Figure 3A, upper/lower panels), each animal serving as its own control. At 192-h post-injection (p.i.), the biodistribution of both particle tracers was also assessed (Figure 3B and Table S4, Supporting Information). At early time points (≈3-h p.i.), dominant cardiac activity (i.e., 16.0 ± 2 %ID/g) was observed after i.v.-injection of ^89^Zr-DFO-scFv-SG4015-PEG-Cy5-C′ dots, indicating particles were largely confined to the blood pool (Figure S6A and Table S3, Supporting Information). Cardiac activity-concentrations gradually declined with time to 8 ± 1.1 %ID/g and 3.1 ± 0.5 %ID/g at 24- and 72-h p.i., respectively (Figure 3C and Table S3, Supporting Information). Hepatic uptake was also found to decrease from 5.2 ± 0.4 %ID/g (3-h p.i.) to 4.9 ± 0.4 %ID/g (72-h p.i.). Measured hepatic uptake values are also found to be substantially lower than those reported previously for larger-size nanoprobes (i.e., larger than 10 nM).^[72]^ Muscle activity, however, remained relatively constant (i.e., ≈1 %ID/g) over the imaging interval (Figure 3C and Table S3, Supporting Information), with similar trends observed in activities and time-activity profiles derived for heart, liver, and muscle after i.v.-injection of the particle control (Figure 3D; Figure S6B and Table S3, Supporting Information). Dominant bladder uptake was clearly observed at early time points for both particle probes on coronal and axial tomographic PET imaging (Figure S6, Supporting Information).

Early accumulations of ^89^Zr-DFO-scFv-SG4015-PEG-Cy5-C′ dots in NCI-N87 tumors could be seen at 3-h p.i. on cross-sectional PET imaging (arrow, Figure 3A, coronal (top panel) and axial (bottom panel) views; uptake was estimated to be 6.9 ± 1.4 %ID/g, a value slighter higher than that found for liver (5.2 ± 0.4 %ID/g). Unlike the time-activity data observed for the liver (Figure 3C), however, NCI-N87 tumor uptake progressively rose over the next 72 h to an average maximal value of 10.7 ± 0.6 %ID/g (*n* = 3), approaching ≈11 %ID/g by 192 h (Figure 3C). By contrast, maximum tumor uptake observed at 72 h p.i. for ^89^Zr-DFO-Ctr/scFv-SG4015-C′ dots was only 5.7 ± 0.49 %ID/g (Figure 3D), followed by a slight decrease to ≈5.1 ± 0.60 %ID/g by 168 h p.i. The 72-h maximum intensity projection (MIP) PET image (Figure 3A, far right) clearly demonstrates a significantly higher tumor uptake for the HER2 targeted particle probe (upper image) than for the particle control (lower image).

The rational design and precise engineering of particle surface chemistry needed to incorporate up to six functionalities, including immune components, is a necessary requisite for minimizing off-target accumulations while maximizing targeted delivery and uptake to sites of disease.^[73]^ Tumor time-activity profiles for targeted and control particle tracers, illustrated in Figure 3E, demonstrate an approximate factor of two differences between uptake values (*p* < 0.01, 24-h p.i.; *p* < 0.001, ≥48-h p.i.). In addition, maximum target-specific accumulations of ^89^Zr-DFO-scFv-SG4015-PEG-Cy5-C′ dots (i.e., ≈11 %ID/g) met a designated endpoint criterion (i.e., 10–15% ID/g). Moreover, specific enhancements were also observed for the targeted particle probe over an extended time interval (i.e., 24–192-h p.i.); these were statistically significant when compared with those for the control particle tracer, thus meeting an additional critical metric and highlighting the need for our targeted particle probe developments. Tumor-blood (T/B) ratios rose to a maximum of ≈9.4 ± 1.4 for the targeted particle group, as against 4.1 ± 0.4 for the control group (*p* < 0.001; Figure 3F). Similar trends were observed for tumor-to-liver (T/L) and tumor-to-muscle (T/M) ratios. For the targeted particle group, maximum values of T/L and T/M ratios were 2.3 ± 0.3 and 10.6 ± 0.8, respectively, which were roughly twofold higher than those observed for the respective control groups (Figure 3G,H). Average tumor uptake values and specific enhancements measured herein following administration of the targeted drug delivery vehicle represent some of the highest values reported across a variety of tumor types relative to other particle-based therapeutic delivery vehicles.

A biodistribution study was conducted at the termination of the imaging study (Figure 3B and Table S4, Supporting Information). For the targeted particle probe, average tumor uptake values were 10.8 ± 0.78 %ID/g, as against 5.1 ± 1.3 %ID/g for control particles (***p < 0.0001). Uptake values for the liver were as low as 4.6 ± 0.20 %ID/g for both particles; these values are significantly lower than those reported for other therapeutic nanocarriers, for example, ^64^Cu-labeled liposomes (≈90-nm), where the reported %ID/g value was ≈14 %ID/g at 4 h post-injection.^[74]^ Interestingly, near complete clearance of blood pool activity was noted (i.e., ≈0.37 ± 0.16 %ID/g) for both probes, in addition to significantly lower tracer activity in the heart and lungs (i.e., <4% ID/g). Only trace amounts of particle activity were detected in the feces (i.e., 0.4 ± 0.45 %ID/g). Taken together, these PK and biodistribution results suggest that the HER2 targeted C′ dot drug-immune conjugate tracer may serve as an ideal candidate for targeted detection of gastric cancer given its beneficial imaging properties, namely high target-specific accumulation, target-to-background enhancements, and reduced off-target uptake.

Based on the measured ^89^Zr-DFO-scFv-SG4015-PEG-Cy5-C′ dot time-activity data, normal-organ absorbed doses were estimated for the 70-kg Reference Adult using the OLINDA dosimetry program.^[75]^ Radiation dosimetry was found to be favorable (Table S5, Supporting Information), with absorbed doses (rad/mCi) and the effective dose coefficient (rem/mCi) comparable to routinely used ^89^Zr-labeled radiopharmaceuticals.

### 2.7. Dose Escalation Studies

To determine an optimal dose level for therapeutic studies, as well as to estimate the TI for scFv-SG4015-PEG-Cy5-C′ dots, a dose escalation study was conducted in NCI-N87 tumor-bearing mice (Figure S7, Supporting Information, upper panel). Total particle treatment doses, ranging from 0 to 2.5 nmols, were administered as 3 i.v. injections, one every third day beginning at 10 days post-inoculation of tumor cell implantation, when tumor sizes were ≈150–200 mm^3^. For particle treatment doses of at least 0.01 nmoles (*p* = 0.02), statistically, significant differences in normalized tumor volumes were observed over that of control (i.e., saline vehicle). The median lethal dose, or LD_50_, of HER2 targeting scFv-SG4015-PEG-Cy5-C′ dots, was determined to be 2.5 nmol, as this led to a 50% animal mortality rate (black line, Figure S7, Supporting Information). This result corresponded with body weight losses of more than 20% of the original weight (Figure S7, Supporting Information, lower panel). Total particle doses of 1.9 and 1.2 nmol, on the other hand, showed significant (and roughly equivalent) tumor volume reductions over the first 20 days post-treatment, with relatively stable tumor volume reductions seen thereafter for the latter dose.

These findings were accompanied by body weight losses of 10% or less of the original body weight, which was not considered toxic per the study endpoint (i.e., body weight loss > 20% of initial weight). Finally, a median effective dose (ED_50_) of 0.7 nmol was derived (red asterisk, Figure S7, Supporting Information); this is the dose at which the normalized tumor volume decreased by 50% in one-half of the treated animals 4 days after the final particle dose. These results led to the selection of 1.2 nmol as an optimal particle treatment dose for the remaining therapeutic studies. A TI (i.e., LD_50_/ED_50_) of ≈4 was computed for the targeted particle therapy by modeling the normalized tumor volume versus particle dose data 4 days after the final administered dose (see Experimental Section). The TI can be a useful indicator, albeit controversial in terms of its significance, and reflects the selectivity of a drug to elicit the desired effect rather than a toxic one.^[76]^

One definition of drugs with a low or narrow TI has been provided in the FDA regulations^[71]^ as those exhibiting a less than twofold difference in median lethal dose (LD_50_) and median effective dose (ED_50_) values.^[77]^ On this basis, the higher TI value calculated for our dose-finding data suggested that the targeted particle therapy had a wide margin of safety.^[78]^ Additional issues that may confound the utility of the TI include PK and pharmacodynamic variability, adequacy of endpoints, and the possibility that the preclinical TI value may not directly extrapolate to patient populations,^[78]^ however, it may aid in the selection of appropriate drugs for a given therapeutic indication.

### 2.8. scFv-SG4015-PEG-Cy5-C′ Dots Inhibit NCI-N87 Tumor Growth and Confer a Survival Benefit

**Figure 4.**
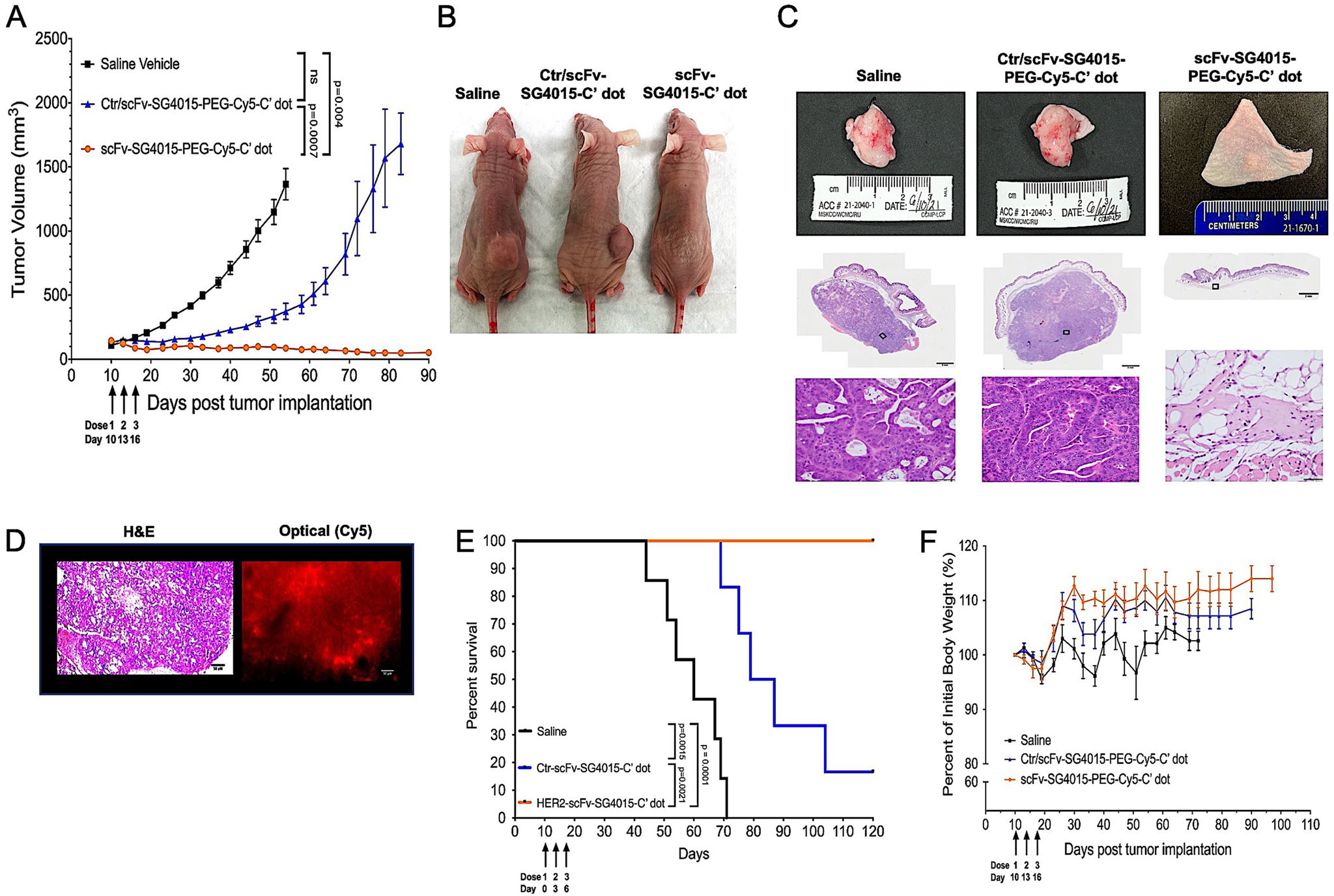
HER2-targeted scFv-SG4015-PEG-Cy5-C′ dots eradicate NCI-N87 tumors and confer a statistically significant survival benefit. A) Tumor growth inhibition curves of NCI-N87 tumor-bearing mice (*n* = 6 per group) i.v.-injected with three doses of saline vehicle, Ctr/svFv-SG4015-PEG-Cy5 C′ dots (isotype control), or scFv-SG4015-PEG-Cy5-C′ dots (HER2 targeted) on days 10, 13, and 16 (total dose: 1.2 nmol). B) Representative mice inoculated with NCI-N87 gastric cancer cells i.v.-injected with the saline vehicle, Ctr/svFv-SG4015-PEG-Cy5-C′ dots (isotype control), or scFv-SG4015-PEG-Cy5-C′ dots(HER2 targeted particles) on days 10, 13, 16 (i.e., total: 1.2 nmol) when tumor sizes were ≈150–200 mm^3^. C) Gross pathology of tumor tissue specimens (upper panel) and matching low- (middle panel) and high-resolution (lower panel) H&E staining, harvested at corresponding treatment endpoints. Scale bars (low-res): 2 mm; (high-res): 50 μm. D) HER2-targeted tissue specimen: H&E (left; scale bar: 50 μm) and Cy5 fluorescence microscopy (right; scale bar: 50 μm) 19 days post-implantation. E) Kaplan–Meier survival curves depicting the overall survival of mice following an identical treatment regimen to (A) (*n* = 6 per group). F) Changes in body weight as a percentage of the initial values (*n* = 6 per group).

To assess the in vivo therapeutic capabilities of scFv-SG4015-PEG-Cy5-C′ dots, NCI-N87 tumor-bearing mice were separated into three treatment groups (*n* = 6 mice per group) as follows: i) scFv-SG4015-PEG-Cy5-C′ dots, ii) Ctr/scFv-SG4015-PEG-Cy5-C′ dots, and iii) saline vehicle. Based on the results of our dose escalation study (Figure S7, Supporting Information), a total particle dose of 1.2 nmol was administered via tail vein injection (i.e., 200 μL per dose, 2 μmol L^−1^) to evaluate growth inhibition, one dose every 3 days, starting at tumor sizes of about 150–200 mm^3^. In a similar manner, mice in the control group were treated with three doses of 200 μL saline vehicle. For each cohort, tumor volumes were monitored by external caliper measurements and animal weights were recorded every 3 days. Statistically significant tumor volume reductions (i.e., >95%) were observed in mice treated with scFv-SG4015-PEG-Cy5-C′ dots, as compared with those treated with Ctr/scFv-SG4015-PEG-Cy5-C′ dots (*p* = 0.0007), persisting 80 days after the first dose was received (Figure 4A). By contrast, animals in the Ctr/scFv-SG4015-PEG-Cy5-C′ dot and vehicle control groups developed tumors larger than 1000 mm^3^ within 55 and 80 days of the first dose received, respectively. In a parallel set of studies, no significant treatment response differences were found in mice triply dosed with particles lacking conjugated SG4015 (i.e., scFv-PEG-Cy5-C′ dots) as against vehicle-treated mice using a similar dose schedule and particle concentrations as in other cohorts (Figure S8, Supporting Information). For all therapeutic studies, animals in which body weight loss was greater than 20% of the original weight or in which tumor masses exceeded 5% of the body weight were sacrificed, and the study was either concluded for that treatment arm or recorded as an event in a Kaplan–Meier survival curve.

At study termination (i.e., 90 days post-implantation), only mice treated with the targeted particle probe showed no evidence of a flank tumor (Figure 4B); this finding persisted in two of the mice up to 195 days post-implantation (data not shown). Harvested tissue specimens from the flanks of each of the three treatment cohorts in Figure 4A are depicted in Figure 4C (upper panel), along with the corresponding low- and high-resolution H&E images (Figure 4C, lower panel). In mice treated with scFv-SG4015-PEG-Cy5-C′ dots, gross examination of H&E-stained sections revealed minimally visible subcutaneous plaque, that, on microscopic evaluation, was fibrotic tissue without evidence of cancer cell infiltration, consistent with complete tumor eradication (Figure 4C). By contrast, excised subcutaneous flank tissues in animals treated with either control particles or saline vehicles were found to harbor numerous cancer cells, consistent with adenocarcinoma. We further evaluated the penetration and distribution of scFv-PEG-Cy5-C′ dots within ex vivo tumor tissue specimens by Cy5 fluorescence microscopy and H&E staining 19 days post-tumor implantation. Fluorescence microscopy confirmed significant tissue penetration, diffusion, and retention of HER2-targeted particles within representative NCI-N87 tissue sections (Figure 4D), suggesting their probable distribution among intracellular and interstitial compartments.

**Figure 5.**
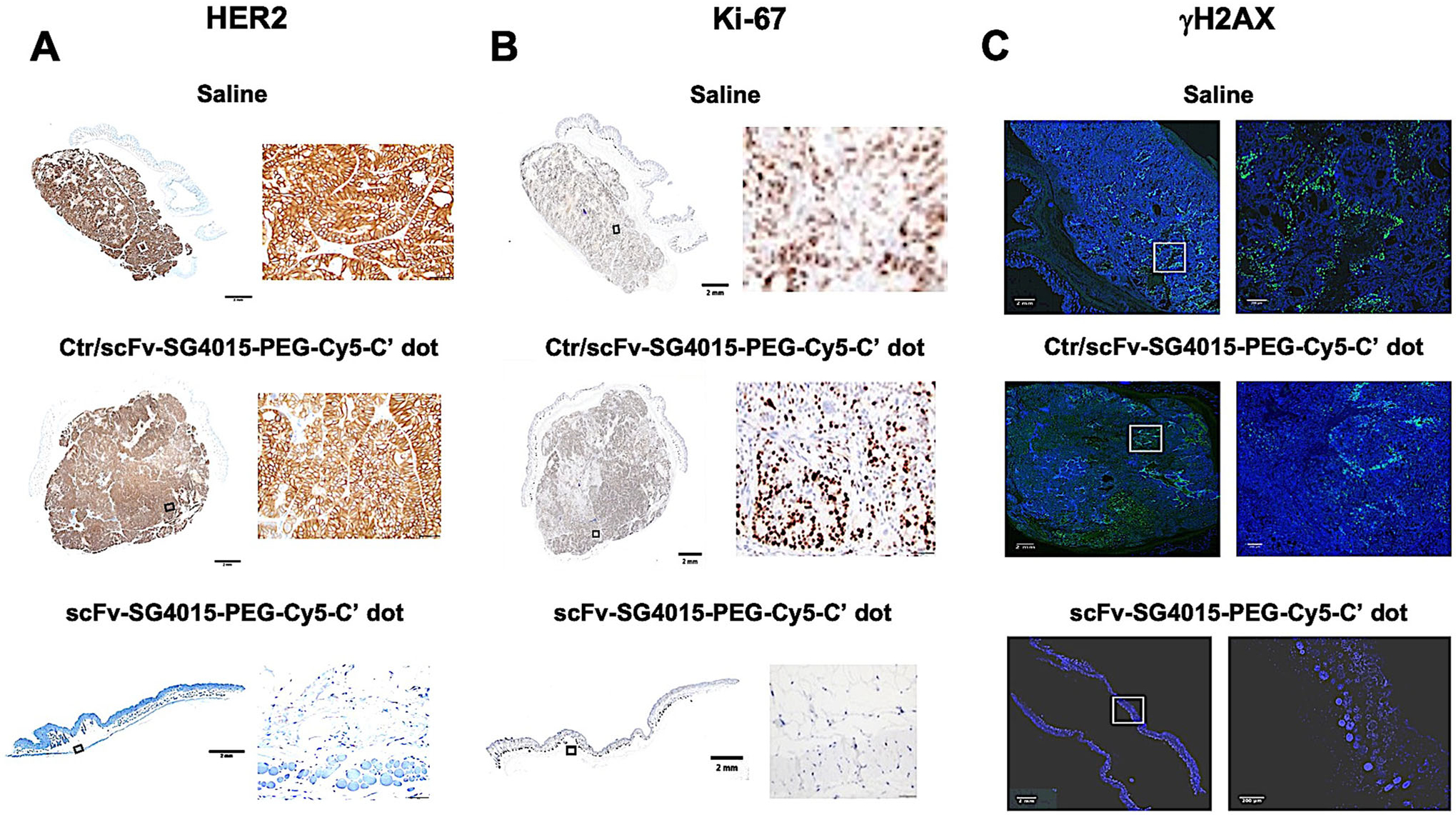
Absence of functional markers in specimens treated with HER2 targeting C′ dots: correlative histology at 90 days post-implantation. NCI-N87 mice were i.v.-injected with saline or administered therapeutically equivalent doses of Ctr/scFv-SG4015-PEG-Cy5-C′ dot or scFv-SG4015-PEG-Cy5-C′ dot for the assessment of HER2 expression, proliferation, and DNA damage in targeted tumor tissues. A) Representative staining of HER2 (brown) by IHC. B) Representative IHC staining of Ki-67 (brown) in fixed tumor tissues. C) Representative IF staining of *γ*H2AX expression (green) in treated tissues with DAPI (blue) counterstaining. Scale bars (all markers; low-res): 2 mm; (Ki-67, HER2; high-res): 50 μm; (*γ*H2AX, high-res): 200 μm.

**Figure 6.**
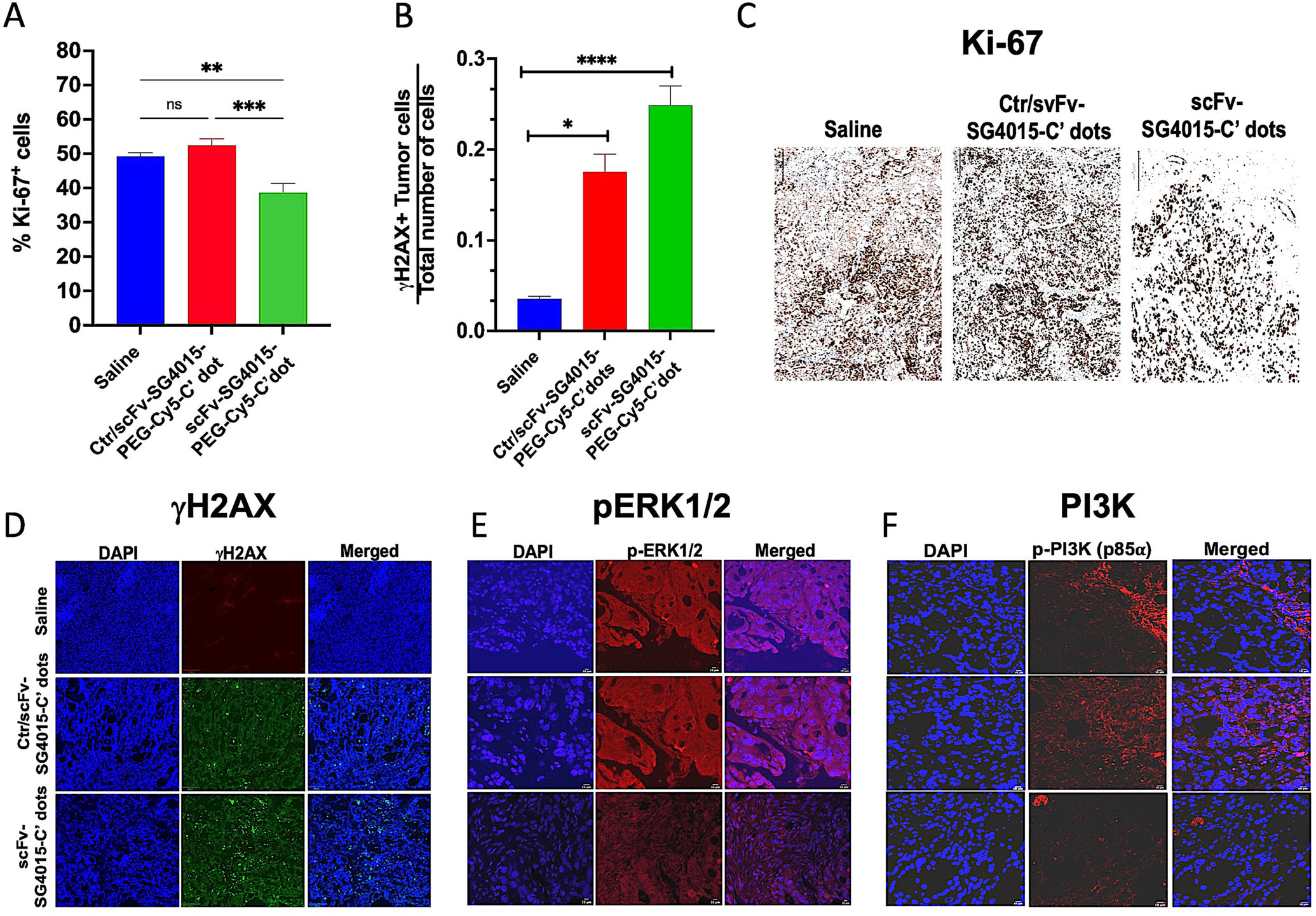
Modulation of functional markers and signaling pathways in tumors exposed to anti-HER2 targeting C′ dots: 30 days post-implantation. Using the same procedures as in Figure 5, tissues were harvested from saline-, Ctr/scFv-SG4015-PEG-Cy5-C′ dot-, and scFv-SG4015-PEG-Cy5-C′ dot-treated mice (*n* = 3 mice/treatment) at 30-days post-implantation to quantitate: A) cellular proliferation (Ki-67) and B) DNA damage (*γ*H2AX). C,D) Corresponding IHC and IF images, repectively, are shown for representative animals: C) Ki-67 (brown; scale bar = 200 μm) and D) *γ*H2AX (green; scale bar = 1 μm). E,F) Representative IF images of signaling pathway intermediates: E) phospho-ERK1/2 (pERK1/2) signaling (red; scale bar = 10 μm) and F) phospho-PI3K p85*α* (red; scale bar = 10 μm). All IF specimens were counterstained with DAPI. Statistical comparisons between the experimental groups were performed by one-way ANOVA followed by a post-hoc Tukey’s test (*p < 0.05; **p < 0.01; ***p < 0.001, ****p < 0.0001).

In addition to tumor growth inhibition studies, we also evaluated whether scFv-SG4015-PEG-Cy5-C′ dots conferred a survival advantage in this gastric cancer model relative to Ctr/scFv-SG4015-PEG-Cy5-C′ dots and/or vehicle control. NCI-N87 tumor-bearing mice were randomly assigned to one of three treatment groups (*n* = 6 per group) using identical dosing regimens to those prescribed for growth inhibition studies. Surprisingly, for the scFv-SG4015-PEG-Cy5-C′ dot group, all mice survived without evidence of tumor regrowth at the termination of the study (i.e., 90 days post-implantation), and yielded statistically significant median survival times compared with animals assigned to either the Ctr/scFv-SG4015-PEG-Cy5-C′ dot (median OS: 83 days, *p* = 0.0021) or vehicle control (median OS: not reached versus 60 days, *p* = 0.001) groups (Figure 4E). It should be noted that while tumors treated with Ctr/scFv-SG4015-PEG-Cy5-C′ dots did not show significant growth inhibition, unlike those treated with targeted particles, tumor progression was delayed, which translated into a significant survival benefit relative to the vehicle control group (median OS: 83 days versus 60 days, *p* = 0.0015). Measured time-dependent differences in the magnitude and rate of tumor volume loss, as well as median survival times, between scFv- and Ctr/scFv-SG4015-PEG-Cy5-C′ dot groups were attributed to an efficient “hit and run” tumor-specific delivery process designed to enhance uptake, retention, and selective cytotoxic/inhibitory responses in NCI-N87 cells while exhibiting bulk renal clearance. Tumor growth inhibition was also observed to be operative through the downregulation of several key signaling pathways modulating HER2 expression (i.e., MAPK/PI3K)^[9,79]^ and other functional tumor markers, including those reflecting proliferative activity (Ki-67) and DNA damage responses (i.e., *γ*H2AX) (Figures 5 and 6).

### 2.9. Toxicology Assessments

Limited toxicology analyses were conducted by an independent pathologist in representative mice from each treatment arm of our growth inhibition study (1.2 nmol), as well as from selected cohorts of our dose escalation study (i.e., 1.2 nmol, 2.5 nmol, saline vehicle). There was no significant loss of body weight (Figure 4F) or dose-limiting toxicity (Tables S5–S9, Supporting Information) found for cohorts treated with 1.2 nmol of scFv-(or Ctr/scFv)-SG4015-PEG-Cy5-C′ dots. These findings contrast with known toxicological sequelae (e.g., hematologic and gastrointestinal) often observed with topoisomerase inhibitors,^[80]^ which have limited their clinical utility as anti-cancer monotherapies. No obvious pathology was found on H&E-stained tissue specimens nor on gross histopathological examination (Table S9, Supporting Information) in mice treated with a 1.2 nmol particle dose. In addition to histopathology, serum chemistries (Table S6, Supporting Information) and complete blood counts (Table S7, Supporting Information) were found to be within normal limits for all treatment groups, with the exception of reticulocyte counts, which were elevated in all cohorts, including vehicle controls, therefore, not related to particle administration. At the LD_50_ dose level (i.e., 2.5 nmol), however, histopathologic changes were found to only affect the small and large bowel (Table S9, Supporting Information, columns 2, 3), probably due to enhanced sensitivity of rapidly dividing cell populations to the topoisomerase inhibitor.

### 2.10. Ex Vivo Histological Analyses of Tumor Tissues

Following efficacy studies, treatment response markers were analyzed in tumor tissues harvested from each cohort 30 days post-implantation and at the termination of the study (i.e., ≈90 days post-implantation). Tumor (HER2), proliferative (Ki-67), and DNA damage (*γ*H2AX) markers were selected based on the mechanism of action for topoisomerase inhibitors. No HER2 staining of tumor tissue specimens treated with scFv-SG4015-PEG-Cy5-C′ dots was seen at study termination by IHC (Figure 5A), as specimens were fibrotic in nature. By contrast, significantly increased HER2 staining was seen at study termination in specimens treated with Ctr/scFv-SG4015-PEG-Cy5-C′ dots or vehicle control (Figure 5A) and was assigned a HER2 score of 3+ on IHC (Figure S9A, Supporting Information).

Ki-67 IHC staining and *γ*H2AX immunofluorescence (IF) studies were also performed at ≈90 days post-implantation (Figure 5B,C) and quantitated by image analysis using positive cell detection algorithms in Qupath.^[81]^ The percentage of Ki-67 positive cells in both control particle- and vehicle-treated tissues was about 63% and 46%, respectively (Figure S9B,C, Supporting Information), while for targeted particle-treated fibrotic tissues, no Ki-67 staining was observed. Finally, the proportion of *γ*H2AX positive tumor cells (i.e., number of *γ*H2AX+ cells divided by the total number of cells) was found to be 0.32 for the control particle, as against 0.14 for vehicle control (Figure S9B,C, Supporting Information), while no *γ*H2AX+ staining was observed in targeted fibrotic particle-treated tissues. These results confirm that, as opposed to saline vehicle control and non-targeted (Ctr/scFv-) C′ dots, the targeted (scFv-) C′ dots effectively eradicated tumor cells and their functional markers.

Statistically significant changes were also seen in the percentage of Ki-67 positive cells and proportion of *γ*H2AX expressing tumor cells among these cohorts at earlier (i.e., 30 days) post-implantation time points (Figure 6A,B). Specifically, for Ki67 (Figure 6A), a 26% loss (*p* < 0.001) of proliferative activity was found between targeted- and control particle-treated tissues, while a similar decrease (i.e., ≈21.4%, *p* < 0.01) was observed between targeted particle and vehicle-treated tissues based on analysis of IHC imaging (Figure 6C). On the other hand, IF images (Figure 6D) showed an increase in the proportion of *γ*H2AX-expressing cells (i.e., ≈79%, *p* < 0.5, Figure 6B) between control particle- and vehicle-treated tissues; this number rose to ≈86% (*p* < 0.0001) for targeted particle treatment. Finally, IF images of representative tumor tissue specimens stained with antibodies against ERK1/2 and PI3K signaling pathway intermediates (Figure 6E,F) showed appreciable decreases in the expression levels of these activation markers, that is, *p*-ERK1/2 (Figure 6E, red) and *p*-PI3K (p85*α*) (Figure 6F, red) for targeted particle-treated specimens, as against specimens treated with either control particles or saline vehicle. Although no changes in HER2 expression were seen at this early time point (Figure S9, Supporting Information, table, column 1), the decrease in these essential pathway markers downstream of HER2 suggests that there is inhibition of HER2 signaling (i.e., tumor cell proliferative capacity, survival). These findings implicate a dual mechanism of action, namely, that while key signaling pathways are inhibited as a consequence of HER binding, preventing HER dimerization, there is concomitant upregulation of *γ*H2AX from topoisomerase inhibition.^[82,83]^ Post-translational modifications (i.e., phosphorylation) of the HER2 protein may also be modulated and remain an active area of investigation.

## 3. Conclusion

The results of our therapeutic studies met key endpoint criteria at substantially lower particle concentrations (i.e., 2 μmol L^−1^) than previously utilized in our non-targeted theranostic work.^[54,55]^ Using a “hit and run” delivery approach for the HER2-targeted drug-immune conjugate particle therapy, statistically significant (*p* < 0.05) improvements in tumor regression were observed over the non-targeted therapy with subsequent eradication, confirmed by histopathology. Second, the targeted therapy was well-tolerated demonstrating no dose-limiting toxicity at optimized particle doses, informed by a rigorous dose-finding strategy. Third, quantitation of the therapeutic index suggested a wide margin of safety, that is, high tolerance to a dose increase beyond the ED_50_.^[84]^ Finally, high penetration, accumulation, and distribution of targeted particles within tumor tissue specimens were observed. Contributing factors to these favorable outcomes were multi-fold, including utilization of i) a potent, multivalent, and molecularly targeted ultrasmall particle therapy, ii) site-engineered anti-HER2 scFv fragments, iii) a highly cytotoxic drug, iv) favorable PK, and v) high DPR. Taken together, the results of these therapeutic studies, coupled with histological, metabolic, and hematological parameters, suggest that surface conjugation of the SG4015 toxin and anti-HER2 scFv to C′ dots with a DPR = 40 resulted in a well-tolerated C′ dot drug-immune conjugate exhibiting favorable PK, marked reductions in tumor burden, and prolonged survival that yields a clinically promising translational platform for treating HER2 expressing tumors.

## 4. Experimental Section

### Molecular Biology and Construction of Expression Vectors:

General molecular biological techniques were conducted as previously described.^[85]^ An scFv directed against the extracellular domain of HER2 was based on the mouse antibody 4D5. The variable regions in a VL-VH orientation were generated by gene synthesis. A G4S1 linker was used as a spacer between VL and VH. Amber stop codons were introduced in-frame at pre-selected sites of the light chain (N-terminal and LC100) or heavy chain (C-terminal and HC44) to designate the sites for non-natural amino acid (nnAA) incorporation. nnAA was inserted at N-terminal and C-terminal sites, whereas amino acid substitutions were used at HC44 and LC100. An scFv directed against human metapneumovirus58 was generated as an isotype control antibody.^[86]^ scFv genes were synthesized and assembled in a VL-VH orientation, incorporating an intragenic amber stop codon at position HC44. All scFv genes were cloned under the control of a cytomegalovirus (CMV) promoter in a proprietary vector useful for stable expression in Chinese Hamster Ovary (CHO) cells.

### Generation of Stable Expressors and scFv-AzK Generation:

Stable expression cell lines were used for the production of scFvs containing amino acid derivative N_6_-((2-azidoethoxy) carbonyl)-L-lysine (AzK). scFv expression vectors were linearized by restriction enzyme digestion and transfected into engineered CHO cells stably expressing *M. mazei* pylRS/tRNA (C13–43)^[87]^ using the Amaxa Nucleofector II device, following the manufacturer’s recommendations. Briefly, 5 × 10^6^ cells were transfected with 5 μg of linearized endotoxin-free DNA encoding the target scFv, and cells grown in CD-CHO medium (Life Technologies) supplemented with 50 μM methionine sulfoximine (MSX, Sigma-Aldrich CAT#M5379) in 24-well plates. scFv expression was assessed in surviving cells using a 12-day fed-batch fermentation where cells were grown to a density of 1 × 10^6^ cells mL^−1^ in an AstraZeneca proprietary medium and 2 mm AzK and growth supplements were added to the culture on day 4. The expression levels of the scFv in each culture were assessed by biolayer interferometry using Octet His2 Biosensors (Sartorius). Stable hosts were ranked based on scFv expression levels and the hosts with the highest scFv titers were isolated, banked, and expanded for scFv scale-up and material generation using a fed-batch fermentation as previously described.^[87]^ scFvs were affinity-purified directly from the expression supernatant using His Trap Excel (Millipore Sigma) equilibrated in PBS (pH 7.2), washed with 15 mm imidazole in 25 mm sodium phosphate (NaPi), 0.5 m NaCl pH 7, and eluted in the same buffer containing 300 mm imidazole. Eluted protein was dialyzed in PBS, aggregates removed by size exclusion chromatography using a HiLoad 26/600 Superdex 75 pg column in PBS, and peak fractions concentrated to >5 mg mL^−1^ using a Vivaspin (Sartorius) ultracentrifugation device. Purified scFv was formulated in 25 mm histidine and 150 mm NaCl pH 6.

### Synthesis and Characterization of SG4015 Containing an Enzymatically-Cleavable Linker:

A five-step synthesis was used to generate the payload SG4015, which consisted of an efficacious topoisomerase I inhibitor containing a Val-Ala cleavable dipeptide linker and an azide moiety for enabling azide-alkyne conjugation chemistry. SG4015 was synthesized at AstraZeneca, and the structure was confirmed by mass spectrometry (ESI^+^-MS: *m/z* calculated for C_39_H_48_N_8_O_10_; 788.35; found 790.0 [*M* + H]^+^) and ^1^H NMR.

### Synthesis of DFO-scFv (or Ctr/scFv)-SG4015-PEG-Cy5-C′ Dots:

DFO-DBCO-PEG-Cy5-C′ dots were synthesized and characterized according to previously published procedures.^[63]^ For a typical synthesis of DFO-scFv-SG4015-PEG-Cy5-C′ dots having a DPR of ≈40, DFO-DBCO-PEG-Cy5-C′ dots were first mixed with azido-scFv at a ratio of 1:5 and reacted at room temperature for 2–4 h; then azido-SG4015 was added at a ratio of 1:50 and the mixture kept at room temperature under shaking (650 rpm) for overnight. The unreacted free azido-scFv and azido-SG4015 were purified by SEC chromatography column with Sephadex G-50 medium and purity was determined with a reversed-phase C-18 column on HPLC. As synthesized DFO-scFv-SG4015-PEG-Cy5-C′ dots were then characterized in terms of their hydrodynamic diameter, surface chemical properties, and drug-to-particle ratios using a combination of TEM, FCS, and UV/vis spectroscopy. Similar procedures were used for the synthesis of DFO-DBCO-Ctr/scFv-PEG-Cy5-C′ dots.

### ^89^Zr Production and Radiolabeling of DFO-scFv (or Ctr/scFv)-SG4015-PEG-Cy5-C′ Dots:

^89^Zr was produced on a TR19/9 cyclotron (Ebco Industries Inc.) via the ^89^Y(p,n)^89^Zr reaction, and purified to yield ^89^Zr with a specific activity of 5.28–13.43 mCi μg^−1^ (470–1195 Ci mmol^−1^).^[88]^ Previously established reaction protocols were followed for ^89^Zr radiolabeling.^[57,63]^ Briefly, 1 nmol of scFv- or Ctr/scFv-conjugated DFO-SG4015-C′ dots were mixed with 1 mCi of ^89^Zr-oxalate in HEPES buffer (pH 8) at 37 °C for 60 min; final labeling pH was maintained at 7–7.5. The reaction was followed by an EDTA challenge step at 37 °C for 60 min to remove any non-specifically bound ^89^Zr. Radiolabeled particle products were purified using a PD-10 column, and final yields were measured with a CRC-55TR dose calibrator. ^89^Zr labeling yields were in the range of 70% to 80% (*n* > 5). Radiochemical purity was estimated to be greater than 99% by radio-TLC.

### Stability of DFO-scFv-SG4015-PEG-Cy5-C′ Dots:

For stability studies, 100 μL (15 μM) of DFO-scFv-SG4015-PEG-Cy5-C′ dots (DPR ≈40) were initially evaluated in PBS. The area under the curve (AUC) ratio between SG4015 (AUC_360nm_) and C′ dots (AUC_651nm_) was acquired using well-established HPLC analytical approaches.^[54]^ The percentage (%) of retained SG4015 in DFO-scFv-SG4015-PEG-Cy5-C′ dots was calculated using the following equation (take *t* = 24 h for example)

(1) $$\%\text{retained SG4015}=\frac{\frac{{\text{AUC}}_{360\;\text{nm}(t=24\;\text{h})}}{{\text{AUC}}_{651\;\text{nm}(t=24\;\text{h})}}}{\frac{{\text{AUC}}_{360\;\text{nm}(t=0)}}{{\text{AUC}}_{651\;\text{nm}(t=0)}}}*100$$

Stability was also assessed in serum using UV–vis spectroscopy after mixing 100 μL (15 μM) of DFO-scFv-SG4015-PEG-Cy5-C′ dots in PBS with 100 μL of human or mouse serum and maintained at 37 °C under shaking conditions (650 rpm) for 144 h.

### Cell Culture:

Human NCI-N87 gastric carcinoma and MDA-MB-231 breast cancer cell lines were obtained from American Type Culture Collection (ATCC). Cells were maintained in Roswell Park Memorial Institute media (RPMI 1640), supplemented with 10% fetal calf serum, and 1% penicillin/streptomycin (Media Preparation Facility, Memorial Sloan Kettering).

### In Vitro Particle Uptake, Blocking, and Competitive Binding Studies:

HER2 receptor binding studies were performed by incubating 1.5 ×10^5^ NCI-N87 or MDA-MB-231 cells with either DFO-scFv- (or Ctr/scFv)-SG4015-PEG-Cy5-C′ dots over a range of concentrations (i.e., 10, 20, 50, and 100 nM) for 1 h at 4 °C. Samples were centrifuged, washed with flow buffer, and counterstained with DAPI for dead cell exclusion prior to analysis. In a similar manner, blocking studies were performed by co-incubating DFO-scFv- (or Ctr/scFv)-SG4015-PEG-Cy5-C′ dots with free scFv-488 at 100 nM and 2μM concentrations, respectively. Competitive binding studies were performed by co-incubating fixed concentrations of free scFv-488 (40 nM) with increasing concentrations of DFO-scFv-SG4015-PEG-Cy5-C′ dots. Briefly, triplicate samples (1.5 × 10^5^ NCI-N87 or MDA-MB-231 cells per tube) were incubated with appropriate concentrations of free scFv-488 and DFO-scFv-SG4015-PEG-Cy5-C′ dots for 1 h at 4 °C. Following incubation, tubes were centrifuged and washed three times in a cold flow cytometry buffer. Samples were counterstained with DAPI prior to analysis for dead cell exclusion. In both assays, samples were analyzed on a BD LSRFortessa flow cytometer (BD Biosciences, San Jose, CA). Results were displayed as MFI using Prism9 software (GraphPad). For competitive binding assays, MFI values were plotted against log-transformed DFO-scFv-SG4015-PEG-Cy5-C′ dot concentrations, and IC_50_ values were calculated using a sigmoidal 4PL regression analysis with Prism9 software.

### Determination of LD_50_:

LD_50_ values were determined by incubating cells with increasing concentrations of either SG4015 toxin or scFv-SG4015-PEG-Cy5-C′ dots in opaque 96-well plates. Briefly, NCI-N87 cells were plated at a density of 5 × 10^3^ cells per well and allowed to attach overnight in full-growth media. Next, cells were treated with free SG4015 toxin or scFv-SG4015-PEG-Cy5-C′ dots containing media for 7 days. At study termination, cell viability was measured using the CellTiter-Glo assay (Promega). LD_50_ values were estimated using a sigmoidal 4PL regression analysis,^[63]^ and displayed using Prism9 software (GraphPad).

### Super-Resolution Confocal Microscopy:

The uptake of scFv-SG4015-PEG-Cy5-C′ dots relative to Ctr/scFv)-SG4015-PEG-Cy5-C′ dots was compared using NCI-N87 and MDA-MB-231 cells. Cells were plated on 8-well micro-slides (Ibidi) at a density of 1 × 10^4^ cells per well, permitted to attach to slides overnight, and then treated with full growth media supplemented with scFv-(or Ctr/scFv)-SG4015- PEG-Cy5-C′ dots (100 nM) for 24 h. Cells were washed 2× with PBS, counterstained with 0.05 mg mL^−1^ Hoechst in PBS for 15 min, and imaged using a Zeiss LSM880 point-scanning confocal microscope equipped with an Airyscan, super-resolution detector (Molecular Cytology Core, Memorial Sloan Kettering Cancer Center, New York, NY). Images were processed using Zen software (Zeiss) and displayed using Imaris Image Analysis Software (Bitplane).

### Animal Models:

All animal studies were performed in accordance with protocols approved by the MSKCC Institutional Animal Care and Use Committee and conformed to NIH guidelines for animal welfare (Protocol Number 12-07-013). NCI-N87 xenografts were established in 6–8-week-old female athymic nu/nu (nude) mice. Mice were inoculated subcutaneously in the right flank with 2 × 10^6^ viable cells using 100 μL of a 1:1 mixture of Matrigel and PBS. Tumor growth was monitored and recorded via caliper measurements. In vivo imaging and particle treatment studies were conducted once tumors attained a size of 150–200 mm^3^.

### In Vivo PET Imaging and Biodistribution Studies in NCI-N87 Mice:

For in vivo PET imaging, mice bearing NCI-N87 flank tumors were i.v.-injected with 200–300 mCi (7.4–11.1 MBq) of ^89^Zr-DFO-scFv (or Ctr/scFv)-SG4015-PEG-Cy5-C′ dot tracers. 5 min prior to PET imaging, mice were anesthetized by inhalation of a 2% isoflurane (Baxter Healthcare)/oxygen gas mixture and placed on the scanner bed; anesthesia was maintained using a 1% isoflurane/gas mixture. Serial PET imaging was performed at 3-, 24-, 48-, 72-, 144-, and 192-h p.i. using a small-animal PET scanner (Focus 120 microPET; Concorde Microsystems). An energy window of 350–700 keV and a coincidence timing window of 6 ns was used. Data were sorted into 2D histograms by Fourier rebinning, and transverse images were reconstructed by filtered back projection into a 128 × 128 × 63 (0.72 × 0.72 × 1.3mm^3^) matrix. PET imaging data were normalized to correct for nonuniformity of response, dead-time count losses, positron branching ratio, and physical decay to the time of injection; no attenuation, scatter, or partial volume averaging corrections were applied. The counting rates in the reconstructed images were converted to activity concentrations (percentage injected dose per gram of tissue, %ID/g) by use of a system calibration factor derived from the imaging of a mouse-sized water equivalent phantom containing ^89^Zr. Region-of-interest (ROI) analyses of the PET data were performed using Inveon Research Workplace (IRW) software; results were presented as %ID/g values. At the last p.i. time point (192 h), mice were sacrificed after scanning, and tumor and major organs were harvested for ex vivo radioassay analysis. Mouse organs were wet-weighted, counted in a Wizard2 g-Counter (PerkinElmer), and converted to %ID/g (mean +/− SD).

### Dosimetry:

Heart, liver, muscle, tumor, and whole-body ^89^Zr time-activity data (percent of the injected dose per gram, %ID/g) were measured by a combination of scintillation well counting of weighed tissue samples excised at necropsy and serial quantitative PET imaging plus ROI analysis. For each tissue and the whole body, exponential functions were fit to the resulting time-activity data (corrected for radioactive decay to the time of injection) and analytically integrated, incorporating the effect of radioactive decay, to yield the respective ^89^Zr time-integrated activity coefficients per gram of tissue (mCi-h/g/mCi). The mean absorbed doses (rad/mCi) in mice were then calculated by multiplying the time-integrated activity coefficients per gram by the ^89^Zr equilibrium dose constant (g-rad/(mCi-h)) for non-penetrating radiations (positrons), assuming complete local absorption of such radiations and ignoring the contribution of penetrating radiations (i.e., gamma-rays). For the human dosimetry, the mouse time-integrated activity coefficients per gram were converted to the corresponding time-integrated activity coefficients (mCi-h/mCi) for the 70-kg standard adult male anatomic model, taking into account differences in whole-body and organ masses between mice and the 70-kg standard adult male. The resulting human time-integrated activities were reexpressed in units of mCi-h/mCi or simply *h* (the latter formerly known as the residence time). The human residence time values were then entered into the OLINDA dosimetry program to calculate the mean organ and whole-body absorbed dose coefficients (rad/mCi) and the effective dose coefficient (rem/mCi) using the formalism of the Medical Internal Radiation Dosimetry (MIRD) Committee of the Society of Nuclear Medicine and Molecular Imaging. This human dosimetry formalism model was for “normal” (i.e., tumor-free) anatomy and thus did not yield estimates of tumor doses.^[54,75]^

### Dose Escalation Studies:

Forty-two NCI-N87 tumor-bearing mice were randomized into nine cohorts (*n* = 3–5 mice per cohort) and a triple-dose study was conducted using a saline vehicle or scFv-SG4015-PEG-Cy5-C′ dots, with total particle doses ranging from 0 to 2.5 nmoles (200 μL per injection). Specifically, doses were administered systemically every third day (i.e., days 0, 3, and 6) to the following groups of mice once tumors attained sizes of 150–200 mm^3^ (10 days post-inoculation): Group 1: Vehicle control; Group 2: 0.002 nmol; Group 3: 0.01 nmol; Group 4: 0.02 nmol; Group 5: 0.12 nmol; Group 6: 0.25 nmol; Group 7: 1.2 nmol; and Group 8: 1.9 nmol; and Group 9: 2.5 nmol. Tumor volumes were monitored over time until the point of sacrifice (i.e., 18 days after the final dose), and normalized to the initial volumes measured. An optimal dose for efficacy studies was determined on the basis of the median lethal dose (or LD_50_), or the dose at which 50% of animals experience mortality.^[89]^ In addition, the dose of a drug required to elicit the desired pharmacological effect (i.e., 50% reduction of the maximum tumor volume loss) in 50% of animals, or median effective dose (ED_50_),^[90]^ was determined by fitting a sigmoidal mathematical function (Excel, Microsoft, Redmond, WA) to the dose-response data (i.e., normalized tumor volume vs drug dose) 4 days after the final particle dose. Such functions were commonly used to model pharmacological dose-response data. Based on the fitted sigmoidal function, the ED_50_ was determined and the therapeutic index computed (i.e., the ratio of TD_50_ to ED_50_)^[89]^ for these preclinical studies.

### In Vivo Growth Inhibition and Therapeutic Efficacy Studies:

Following the initiation of NCI-N87 flank xenografts, a multi-dose treatment regimen was initiated once tumors reached ≈150–200 mm^3^ by caliper measurements. In brief, tumor-bearing mice were randomly assigned to receive one of the following three treatments (*n* = 6 mice per group): saline vehicle, scFv-SG4015-PEG-Cy5-C′ dots (1.2 nmol total; 2.5 μM), or Ctr/scFv-SG4015-PEG-Cy5-C′ dots (1.2 nmol total; 2.5 μM). Treatments were i.v.-injected (200 μL) on days 0, 3, and 6 once tumors attained sizes of ≈150 mm^3^ (10 days post-inoculation). Tumor volumes were measured daily via calipers and volumes were calculated using the formula: volume = (long axis × short-axis^2^)/2. For survival studies, NCI-N87 xenografted mice (*n* = 6 per group) underwent identical treatment protocols to those described above, with tumors measured by external calipers daily. For all studies, animals in each treatment arm were monitored over time and sacrificed if one of the following endpoints was reached: i) tumor mass >5% of the body weight or body weight loss >20% of the original weight. Once an animal met one of these established endpoints, the study was either concluded for that treatment arm or was recorded as an event in a Kaplan–Meier survival curve. Data were graphically displayed using Prism9 software (GraphPad).

### Fluorescence Microscopy:

To assess scFv-SG4015-PEG-Cy5-C′ dot distribution in tumor tissues, harvested NCI-N87 tumors were mounted in Tissue-Tek O.C.T. and sectioned at a thickness of 10 μm on an Avantik Cryostatic Microtome (Avantik Biogroup, Springfield Township, NJ). H&E staining of tumor tissue specimens was performed, along with the acquisition of Cy5 fluorescence images using a BX60 fluorescence microscope (Olympus America Inc., Center Valley, PA) equipped with a motorized stage (Prior Scientific Instruments Ltd., Rockland, MA) and CC12 camera (Olympus) at 10× magnification. Images of entire tumor sections were obtained by acquiring data across multiple fields, which were subsequently stitched together using MicroSuite Biologic Suite (version 2.7, Olympus). Brightfield (hematoxylin and eosin or H&E) and fluorescence (Cy5) images were acquired using the appropriate filter cube sets, and fluorescence image processing was carried out using Image J. For quantification of Ki-67 and *γ*H2AX, slides were scanned using a 20×/0.8NA objective on a P250 Panoramic Scanner (3DHistech, Budapest, Hungary), and analyzed using a positive cell detection algorithm in QuPath.^[81]^

### H&E, IHC, and IF Staining for Treatment Response Assessment:

For immunohistochemical (IHC) and IF assays, representative formalin-fixed and paraffin-embedded (FFPE) tumor tissues were sectioned at a thickness of 5 μm on a Discovery XT System (Ventana Medical Systems, Roche, Tucson, AZ). All images were captured using identical acquisition settings and subjected to synonymous post-processing procedures across specimens.

### Ki-67 IHC:

IHC for Ki-67 staining was performed on tumor sections using a Leica Bond RX automated stainer. After heat-induced epitope retrieval in a pH 9 buffer, the primary antibody, rabbit monoclonal antibody clone D3B5 (Cell Signaling # 12 202) was applied at a concentration of 1:500, followed by a polymer detection system, according to the manufacturer’s instructions (DS9800, Novocastra Bond Polymer Refine Detection, Leica Biosystems). The chromogen used was 3,3-diaminobenzidine tetrachloride (DAB), and sections were counterstained with hematoxylin.

### Phospho-H2AX (γH2AX) IF:

IF staining was performed for *γ*H2AX on tumor tissue sections as follows. After heat-induced epitope retrieval in a pH 9 buffer, a primary antibody (anti-*γ*H2Ax 0.2 mg mL^−1^) was applied. Biotinylated goat anti-rabbit IgG secondary antibody was subsequently added (5.75 mg mL^−1^; Vector labs, #PK6101). Application of streptavidin-HRP D (DAB Map kit, Ventana Medical Systems) was followed by incubation with Tyramide Alexa Fluor 488 (Invitrogen, # T20922), prepared according to the manufacturer’s instruction at a 1:150 dilution. Slides were counterstained with DAPI (5 mg mL^−1^, Sigma Aldrich, #D9542) and mounted with Mowiol.

### Phospho-ERK1/2 and Phospho-PI3K (p85α) IF:

IF staining was performed for p-ERK1/2 and p-PI3K on tumor tissue sections. After heat-induced epitope retrieval in a pH 6 buffer, sections were washed twice with buffer (1× TBS/0.1% Tween-20). Endogenous peroxides were blocked with BLOXALL (SP-6000, Vector Labs, Burlingame, CA). Tumor tissues were washed 2× with buffer solution, then incubated with blocking solution (5% Normal Goat Serum/wash buffer) for 1 h at room temperature and washed again. Primary antibodies (anti-pERK1/2 #14-9109-82, Thermo-Fisher, and anti-pPI3K #MA1-74183 Thermo-Fisher at dilutions of 1:250) were applied in blocking solution, and sections incubated overnight at 4 °C in a humidified chamber, followed by multiple washes and addition of Alexa-Fluor 555 conjugated anti-mouse secondary antibody (Cell Signaling, #4412) at a dilution of 1:1000 in blocking solution. Tissue sections were incubated at room temperature for 30 min and washed in triplicate for 5 min. Nuclear counterstaining was performed with ProLong Gold Antifade Mountant with DAPI (Invitrogen, #P36931).

### HER2 Immunofluorescence:

For HER2 IF staining, sections were washed twice with buffer (1× TBS/0.1% Tween-20). Endogenous peroxides were blocked with BLOXALL (SP-6000, Vector Labs, Burlingame, CA). Tumor tissues were washed 2× with buffer solution, then incubated with blocking solution (5% Normal Goat Serum/wash buffer) for 1 h at room temperature and washed again. HER2 (#2165S, Cell Signaling, Danvers, MA) primary antibody (1:400 dilution) in blocking solution was added, and sections incubated overnight at 4 °C in a humidified chamber, followed by multiple washes and addition of FITC conjugated anti-rabbit secondary antibody (Cell Signaling, #4412) at a dilution of 1:300 in blocking solution. Tissue sections were incubated at room temperature for 30 min and washed in triplicate for 5 min. Nuclear counterstaining was performed with DAPI (0.1 mg mL^−1^). Sections were washed twice for 5 min, followed by coverslip application using Prolong Gold Antifade (ThermoFisher, #P10144) mounting media.

### Necropsy and Histopathology:

Following the euthanasia of animals by CO_2_, blood was collected via cardiac puncture immediately following euthanasia. A gross examination was then performed, and organs were fixed in 10% neutral buffered formalin, followed by decalcification of bone in a formic acid solution (Surgipath Decalcifier I, Leica Biosystems). Tissues were then processed in ethanol and xylene and embedded in paraffin in a Leica ASP6025 tissue processor. Paraffin blocks were sectioned at 5 μm, stained with H&E, and examined by a board-certified veterinary pathologist (SM). All organs were processed and examined for animals receiving a lethal dose (i.e., 2.5 nmol) including the heart, lungs, submandibular and mesenteric lymph nodes, liver, kidneys, spleen, bone marrow (sternum, femur, tibia, vertebrae), small and large intestines, and brain. Tumor tissue specimens and a limited set of organs were harvested from representative mice receiving therapeutic doses (1.2 nmol) or saline vehicle, namely the liver, spleen, gallbladder, and kidneys.

### Hematology:

For hematology, blood was collected into tubes containing EDTA (BD Microtainer 02-669-33). Automated analysis was performed on an IDEXX Procyte DX hematology analyzer and the following parameters were determined: white blood cell count, red blood cell count, hemoglobin concentration, hematocrit, mean corpuscular volume, mean corpuscular hemoglobin, mean corpuscular hemoglobin concentration, red blood cell distribution width standard deviation and coefficient of variance, reticulocyte relative and absolute counts, platelet count, platelet distribution width, mean platelet volume, and relative and absolute counts of neutrophils, lymphocytes, monocytes, eosinophils, and basophils.

### Serum Chemistry:

For analysis of serum chemistries, blood was collected in tubes containing a serum separator (BD Microtainer 02-675-185), followed by centrifugation to obtain serum for analysis. Serum chemistry was performed on a Beckman Coulter AU680 analyzer and the concentration of the following analytes was determined: alkaline phosphatase, alanine aminotransferase, aspartate aminotransferase, creatine kinase, gamma-glutamyl transpeptidase, albumin, total protein, globulin, total bilirubin, blood urea nitrogen, creatinine, cholesterol, triglycerides, glucose, calcium, phosphorus, chloride, potassium, and sodium. Na/K and albumin/globulin ratios were calculated.

### Statistics:

All statistical analyses were performed using Prism9 software (GraphPad). Group means and standard errors of the mean (SEMs) were calculated for concentration-dependent particle uptake, changes in the biodistribution profiles, and tumor volumes. For biodistribution studies, statistical comparisons between the experimental groups were performed by using an F-test and pairwise *t*-tests, adjusted for multiple comparisons using the Holm method. A one-way or two-way ANOVA was used for growth inhibition studies (*n* = 6 animals per cohort), dose escalation studies (*n* = 3–5 animals per cohort), and representative histological assessments (*n* = 3 animals per cohort), followed by the use of Tukey’s multiple comparison testing. For all these evaluations, results were considered statistically significant if *p* < 0.05. No less than three replicates were generated per group (*p < 0.05; **p < 0.01; ***p < 0.001) unless otherwise noted. Kaplan–Meier analysis was used to plot survival data and the log-rank (Mantel–Cox) test was used to determine statistical significance for overall survival studies (*n* = 6 animals per cohort).

## Supplementary Material

supinfo

## Data Availability

The data that support the findings of this study are available from the corresponding author upon reasonable request.
