# Supplementary material for: Engineered Ultrasmall Nanoparticle Drug-Immune Conjugates with “Hit and Run” Tumor Delivery to Eradicate Gastric Cancer
Source: Adv Ther (Weinh). Author manuscript; Available in PMC 2024 Mar 1. (PMC10061546; doi:10.1002/adtp.202370009)
Supplement: supinfo [file NIHMS1850724-supplement-supinfo.pdf]

ADVANCED THERAPEUTICS

Supporting Information

for *Adv. Therap.*, DOI 10.1002/adtp.202200209

Engineered Ultrasmall Nanoparticle Drug-Immune Conjugates with "Hit and Run" Tumor Delivery to Eradicate Gastric Cancer

Li Zhang, Virginia Aragon-Sanabria, Anusha Aditya, Marcello Marelli, Tianye Cao, Feng Chen, Barney Yoo, Kai Ma, Li Zhuang, Thais Cailleau, Luke Masterson, Melik Z. Turker, Rachel Lee, Gabriel DeLeon, Sebastien Monette, Raffaele Colombo, Ronald J. Christie, Pat Zanzonico, Ulrich Wiesner, J. Anand Subramony* and Michelle S. Bradbury**

Supporting Information

Engineered Ultrasmall Nanoparticle Drug-Immune Conjugates with "Hit and Run" Tumor Delivery to Eradicate Gastric Cancer

Li Zhang,[1,2†] *Virginia Aragon-Sanabria,*[1,2†] *Anusha Aditya,*[1,2†] *Marcello Marelli,*[3] *Tianye Cao,*[1,2] *Feng Chen,*[1,2] *Barney Yoo,*[2,4] *Kai Ma,*[2,5] *Li Zhuang*[3], *Thais Cailleau*[6], *Luke Masterson*[6], *Melik Z. Turker,*[2,5] *Rachel Lee,*[2,5] *Gabriel DeLeon,*[1,2†] *Sebastien Monette,*[7] *Raffaele Colombo,*[3] *Ronald J. Christie,*[3] *Pat Zanzonico,*[2,8] *Ulrich Wiesner,*[2,5,9*] *J. Anand Subramony,*[3*] *Michelle S. Bradbury*[1,2,10*]

[1]Department of Radiology, Sloan Kettering Institute for Cancer Research, New York, NY 10065, USA
[2]MSK-Cornell Center for Translation of Cancer Nanomedicines, Sloan Kettering Institute for Cancer Research, New York, NY 10065, USA
[3]AstraZeneca, One MedImmune Way, Gaithersburg, Maryland 20878, United States
[4]Department of Chemistry, Hunter College, New York, NY 10065, USA
[5]Department of Materials Science & Engineering, Cornell University, Ithaca, NY 14853, USA
[6]AstraZeneca, Spirogen, QMB Innovation Centre, 42 New Road, London, E1 2AX
[7]Laboratory of Comparative Pathology, Sloan Kettering Institute for Cancer Research, Weill Cornell Medicine, The Rockefeller University, New York, NY 10065, USA
[8]Department of Medical Physics, Sloan Kettering Institute for Cancer Research, New York, NY 10065, USA
[9]Kavli Institute at Cornell for Nanoscale Science, Cornell University, Ithaca, NY 14853, USA
[10]Molecular Pharmacology Program, Sloan Kettering Institute for Cancer Research, New **York, NY 10065, USA**

Table of Contents

Figures S1-S9………………………………………………………………..… 3- 11

Tables S1-S9…………………………………………………………………12-20

A

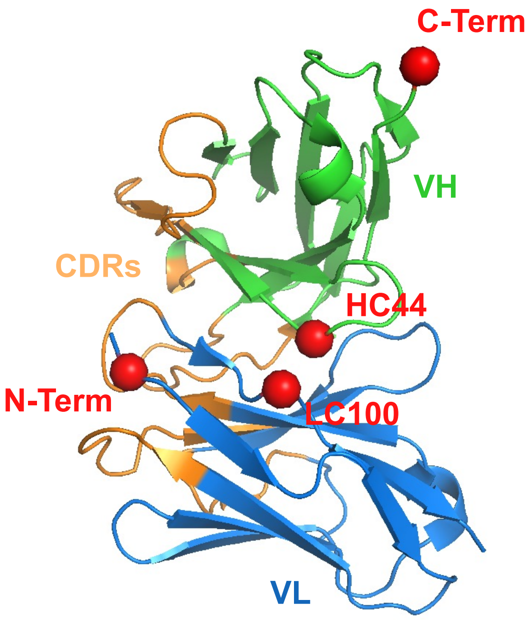

B

```
 N-Term                                       VL CDR1
1   D*IQMTQS PSSLSASVGD RVTITCRASQ DVNTAVAWYQ QKPGKAPKLL

    VL CDR2                                      VL CDR3
51  IYSASFLYSG VPSRFSGSRS GTDFTLTISS LQPEDFATYY CQQHYTTPPT

    VL100           G4S1 Linker
101 FGQGTKVEIK GGGGSGGGGS GGGGSGGGGS EVQLVESGGG LVQPGGSLRL

                VH CDR1      VH44      VH CDR2
151 SCAASGFNIK DTYIHWVRQA PGKGLEWVAR IYPTNGYTRY ADSVKGRFTI

                                  VH CDR3             C-Term
201 SADTSKNTAY LQMNSLRAED TAVYYCSRWG GDGFYAMDYW GQGTLVTVSS*GPPR
```

Figure S1. Three-dimensional scFv model depicting conjugation and active binding sites indicated in the anti-HER2 scFv sequence. (A) Particle conjugation (HC44) and scFv active binding (CDR) sites. The positions evaluated for conjugation (N-Term, VL100, VH44 and C-Term) relative to the antigen binding domains are illustrated. (B) The sequence for scFv is derived from Trastuzumab, with conjugation sites labeled that correspond to those depicted in (A). AzK was inserted at positions marked with an asterisk (N-Term and C-Term); Substitutions of the marked residues with AzK were used at sites VL100 and VH44. VH, variable fragment heavy chain; VL, variable fragment light chain (Kappa isotype); CDR, complementarity-determining regions.

A

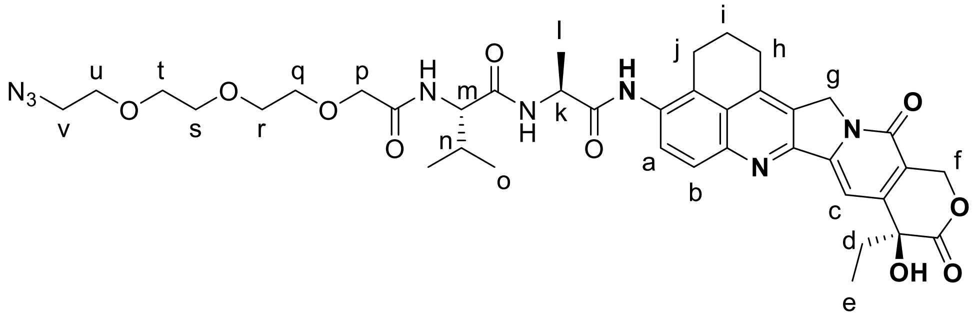

B

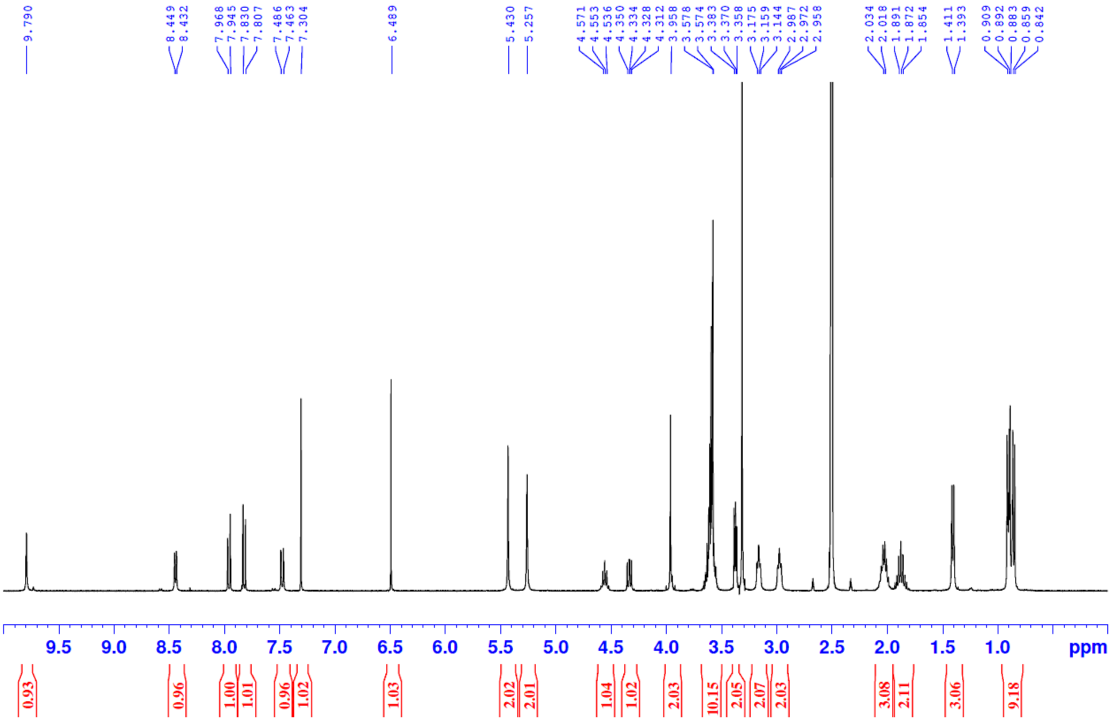

Figure S2. Structure of SG4015.

(A) Atom assignments used for ^{1}H NMR analysis. (B) ^{1}H NMR spectrum and chemical shifts of SG4015 (400 mHz, DMSO-d_6). δ 9.79 (s, 1H, NH aniline), 8.44 (d, *J* = 6.8 Hz, 1H, NH ala), 7.96 (d, *J* = 9.1 Hz, 1H, a), 7.82 (d, *J* = 9.1 Hz, 1H, b), 7.47 (d, *J* = 9.0 Hz, 1H, NH val), 7.30 (s, 1H, c), 6.49 (s, 1H, OH), 5.43 (s, 2H, f), 5.26 (s, 2H, g), 4.55 (dq, *J* = 6.9 Hz, 1H, k), 4.33 (dd, *J* = 9.1, 6.4 Hz, 1H, m), 3.99 – 3.93 (s, 2H, p), 3.66 – 3.51 (m, 10H, q,r,s,t,u), 3.41 – 3.33 (m, 2H, v), 3.16 (t, *J* = 6.4 Hz, 2H, h), 2.97 (t, *J* = 6.2 Hz, 2H, j), 2.02 (p, *J* = 6.6 Hz, 2H, i), 2.02 (m, *J* = 6.6 Hz, 1H, n), 1.87 (q, *J* = 7.2 Hz, 2H, d), 1.40 (d, *J* = 7.1 Hz, 3H, l), 0.94 – 0.81 (m, 9H, e,o).

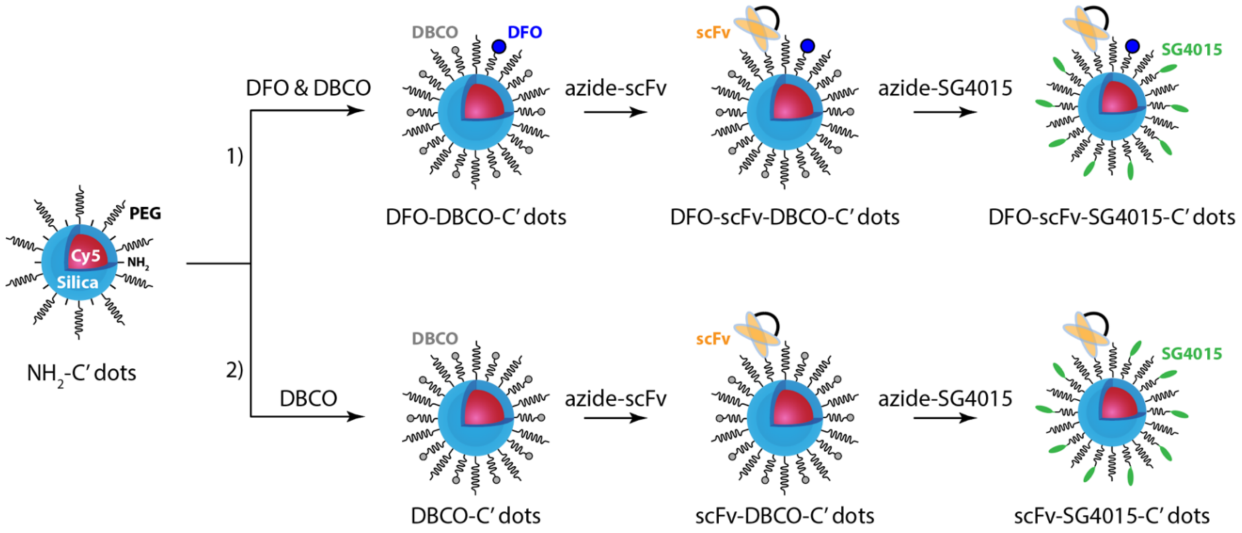

Figure S3. A schematic illustration showing the multiple-step synthesis of scFv-SG4015-C'dots with or without DFO conjugation. The synthesis started with aminated C'dots, NH_2-C'dots. For the synthesis of DFO-scFv-SG4015-C'dots (**Route #1**), the NH_2-C'dot was first reacted with DFO-NCS and DBCO-PEG-NHS ester to form DFO-DBCO-C'dots. After that, anti-HER2 scFv and enzymatically cleavable SG4015 drug linkers were conjugated to the DFO-DBCO-C'dots via strain-promoted azide-alkyne click chemistry (SPAAC) reaction between the DBCO groups on the C'dots and the azide group on the scFv antibody fragment or SG4015 drug linker, respectively. Similar procedures were used for the synthesis of scFv-SG4015-C'dots without DFO chelators (**Route #2**).

Description of the final scFv-SG4015-C'dots with or without DFO conjugation and intermediates shown in Figure S2 using a comprehensive nomenclature[44] and providing more chemistry details (in short: moieties are listed from left to right in the sequence of their synthesis; encapsulated dye in brackets; functional units in bold and italics; moieties connected via a dash are connected to each other, while moieties following an underscore are conjugated directly to the silica core surface rather than to the moiety to the left of it):

Left side:
NH_2-C'dots: C'Dot(***Cy5***)-PEG6-9_amine

Top row:
DFO-DBCO-C'dots: C'Dot(***Cy5***)-PEG6-9_amine-NCS-***DFO***_amine-NHS-PEG4-DBCO
DFO-scFv-DBCO-C'dots: C'Dot(***Cy5***)-PEG6-9_amine-NCS-***DFO***_amine-NHS-PEG4-DBCO-azide-***scFv***_ amine-NHS-PEG4-DBCO
DFO-scFv-SG4015-C'dots: C'Dot(***Cy5***)-PEG6-9_amine-NCS-***DFO***_amine-NHS-PEG4-DBCO-azide-***scFv***_ amine-NHS-PEG4-DBCO-azide-***SG4015***

Bottom row:
DBCO-C'dots: C'Dot(***Cy5***)-PEG6-9_amine-NHS-PEG4-DBCO
scFv-DBCO-C'dots: C'Dot(***Cy5***)-PEG6-9_amine-NHS-PEG4-DBCO-azide-***scFv***_ amine-NHS-PEG4-DBCO
scFv-SG4015-C'dots: C'Dot(***Cy5***)-PEG6-9_amine-NHS-PEG4-DBCO-azide-***scFv***_amine-NHS-PEG4-DBCO-azide-***SG4015***

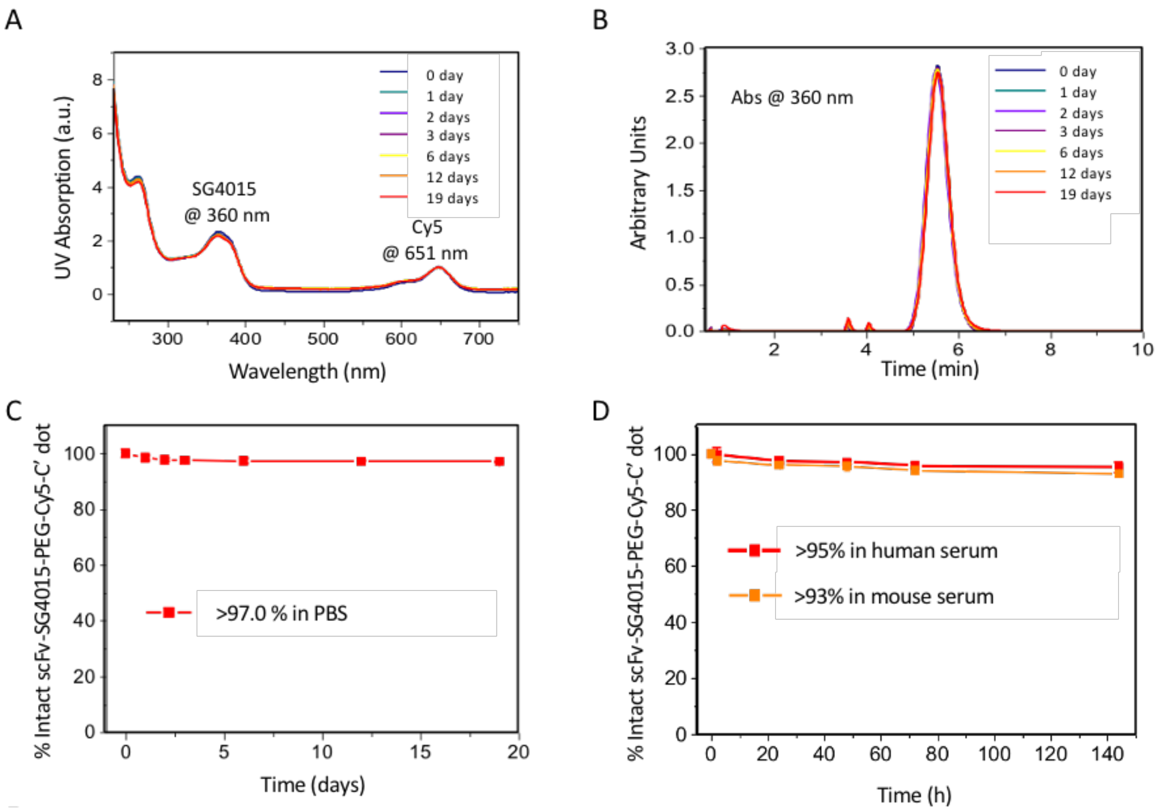

Figure S4. Characterization of scFv-SG4015-PEG-Cy5-C' dots in PBS and serum. (A) Representative UV-Vis spectra of scFv-SG4015-PEG-Cy5-C' dots in PBS (DPR = 40) over a 19-day period post-synthesis with absorption peaks at 360 nm (SG4015) and 651 nm (Cy5-C' dots), respectively. (B) HPLC traces of the scFv-SG4015-PEG-Cy5-C' dot product at 360 nm over 19 days post-synthesis. (C,D) Evaluation of payload stability in PBS (C) and mouse and human sera (D) at 37 ºC under shaking conditions as determined by UV-Vis absorption spectroscopy.

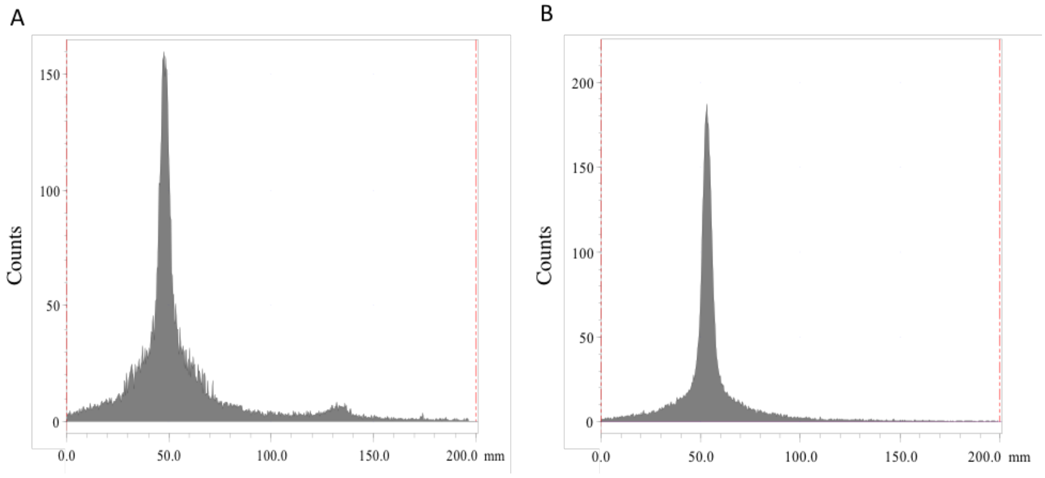

Figure S5. Purity of radiolabeled ^{89}Zr-DFO-scFv-SG4015-PEG-Cy5-C' dot. A-B, iTLC chromatograms of ^{89}Zr-DFO-scFv-SG4015-PEG-Cy5-C' dots before (A) and after (B) purification with a PD-10 column.

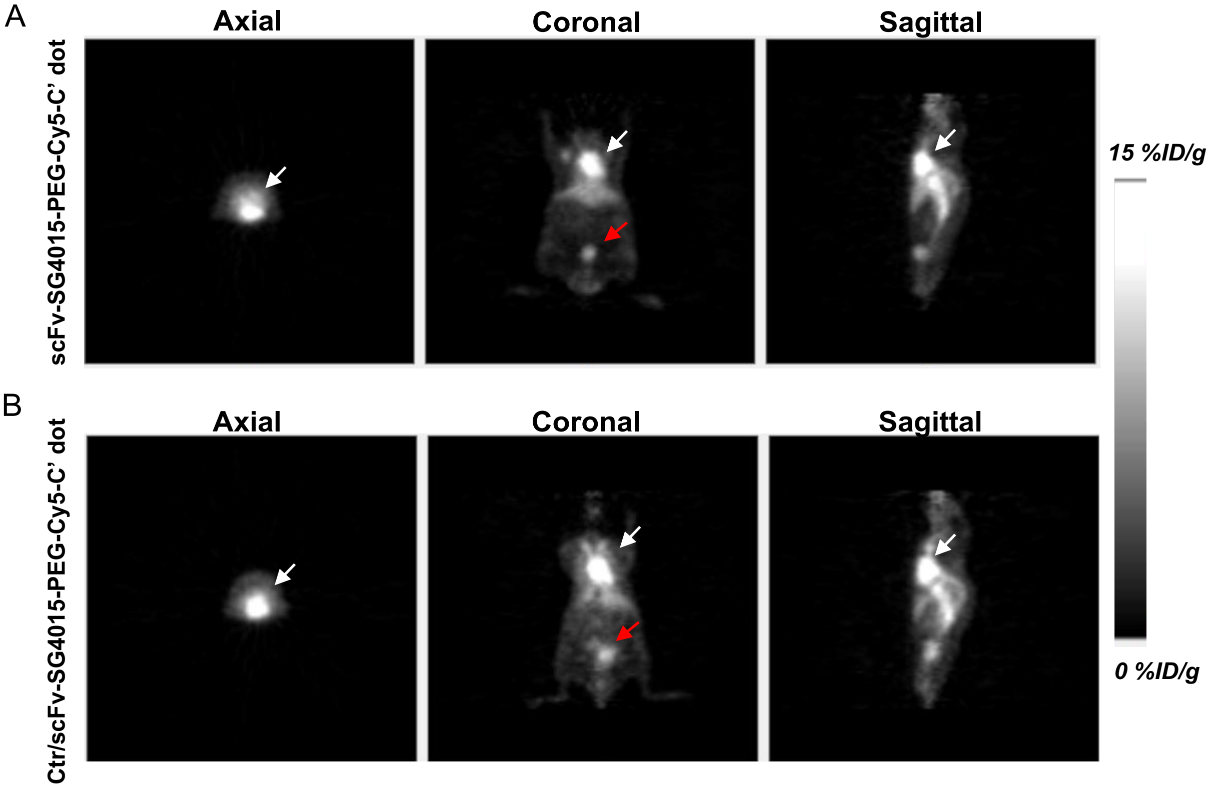

Figure S6. In vivo HER2-targeted PET imaging in a gastric cancer xenograft model. Analysis of coronal, transverse and sagittal tomographic PET images acquired at 3h post i.v. injection of radiolabeled particle immunoconjugates in NCI-N87 mice bearing flank tumors (n = 6 for each group) as follows—targeted group: ^{89}Zr-DFO-scFv-SG4015-PEG-Cy5-C' dots (A) and isotype control group: ^{89}Zr-DFO-Ctr/scFv-SG4015-PEG-Cy5-C' dots (B). Activity in the cardiac blood pool (white arrows) and bladder (red arrows) are indicated for each group.

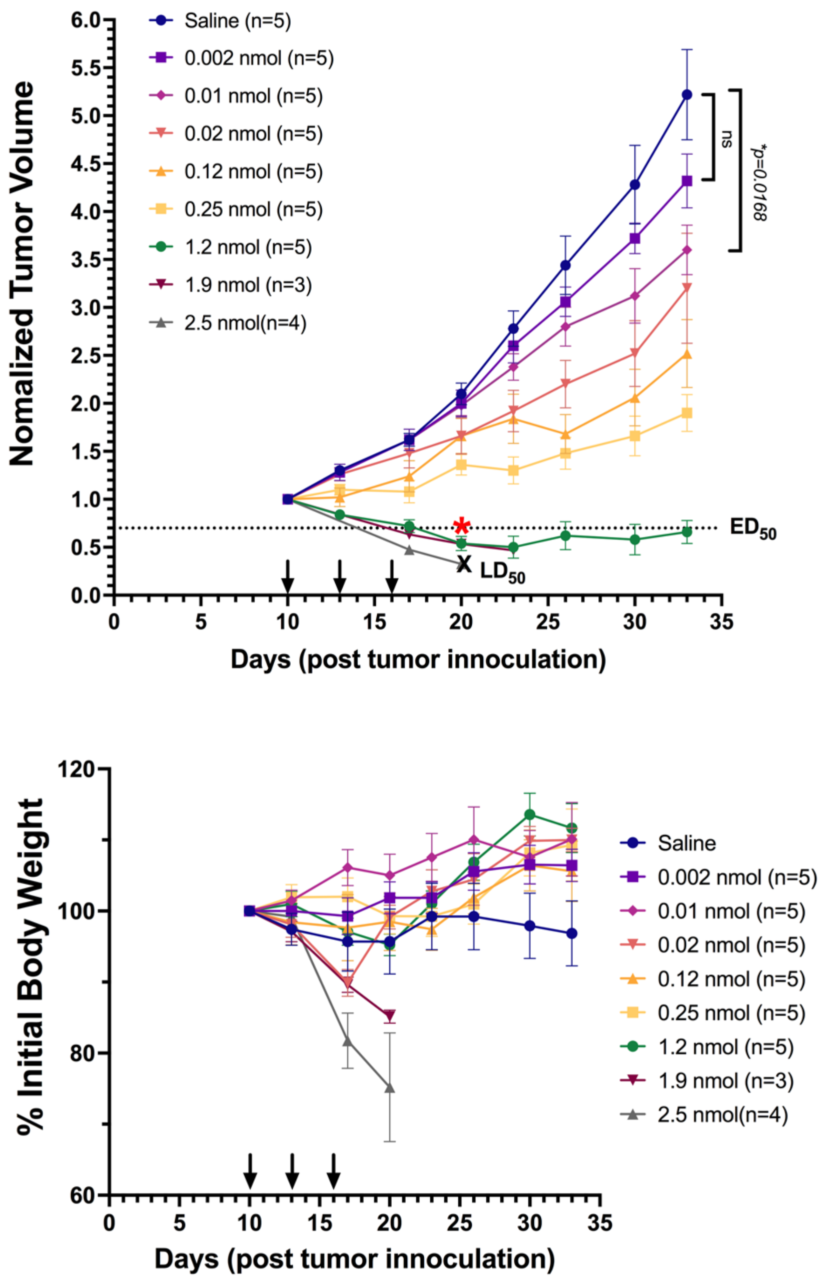

Figure S7. Dose escalation of scFv-SG4015-PEG-Cy5-C' dots in a NCI-N87 gastric cancer model. (*Upper panel*) Total particle dose escalation scheme (range: 0 to 2.5 nmols) performed in tumor-bearing mice (*n*=3-5 mice/group), with doses administered as *n*=3 separate intravenous injections (arrows) every third day beginning at 10 days post-tumor inoculation. Median lethal dose, LD_{50}, and median effective dose, ED_{50}, values were determined and used to compute the therapeutic index. A one-way or two-way ANOVA followed by the use of Tukey's multiple comparison testing was used to establish significance. (*Lower panel*) Corresponding dose-dependent animal weights, as a percentage of the initial body weight.

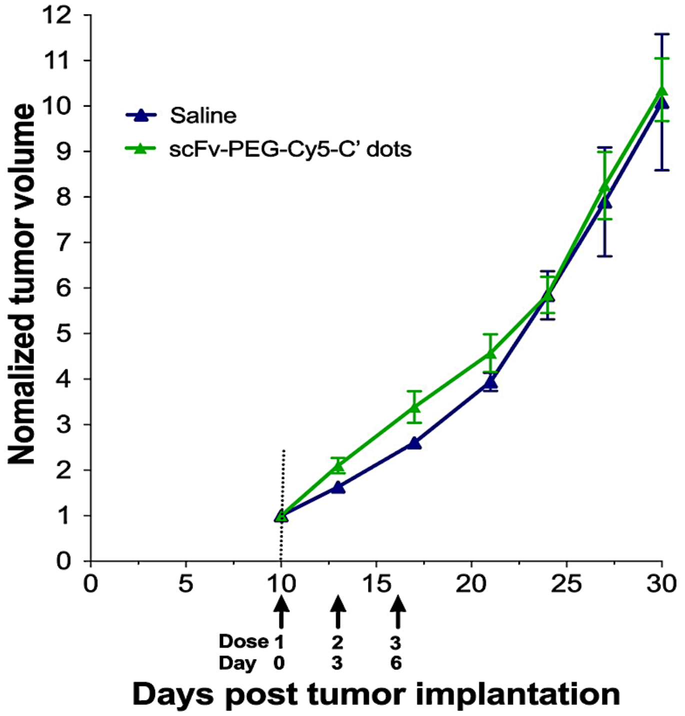

Figure S8. HER2 targeted scFv-PEG-Cy5-C' dots without a SG4015 cytotoxic payload do not lead to tumor growth inhibition. Growth inhibition curves of NCI-N87 tumor–bearing mice (n = 3/group) *i.v.*-injected with a multi-dose (*n*=3) regimen of scFv-PEG-Cy5-C' dots (total dose/concentration: 1.2 nmol / 2μM) or saline vehicle post tumor implantation.

A

Treatment	HER2 score	
	30 days	*96 days*
Saline vehicle	3+	3+
Ctr/scFv-SG4015-PEG-Cy5-C' dot	3+	3+
scFv-SG4015-PEG-Cy5-C' dot	3+	--

B C

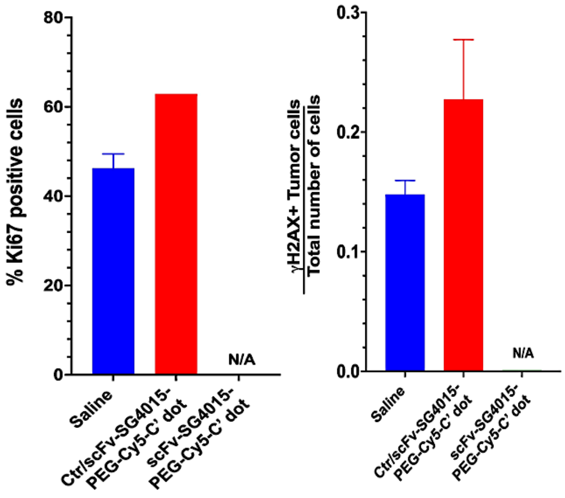

Figure S9. Quantitation of HER2, Ki-67 and γH2AX expression in gastric cancer tissue. HER2 scoring of tumor tissue shown in **Figures 5A-C** (*bottom panel*). Quantitation of the percentage of positive Ki-67 cells in tumor tissue specimens using positive cell detection algorithms in QuPath (*n*=3 mice/cohort). Quantification of the proportion of γH2AX cells over the total number of DAPI-stained cells in tumor (bottom panel) using QuPath software (*n*=3 mice/cohort). N/A, not applicable.

Table S1. FDA approved HER2-targeted antibody or antibody-drug conjugates in advanced gastric cancer patients

Drug Brand	Setting/Phase	Drug Type	Manufacturer	Approval (Yr)	Drug Description
Herceptin®/ Trastuzumab + chemotherapy	1st line Phase 3 (ToGA)	Monoclonal antibody	Roche	1998	Monoclonal antibody used in combination with chemotherapy for treating advanced gastric cancers overexpressing HER2 protein
Pembrolizumab/ trastuzumab/ fluoropyrimidine - and platinum-based chemotherapy	1st line Phase 3 (KEYNOTE-811)	Monoclonal antibodies	Merck & Co. Roche (monoclonal antibodies)	2021	Anti-HER2 (trastuzumab) plus anti-PD1(pembrolizumab) antibodies and fluoropyrimidine- and platinum-based chemotherapies in patients with previously untreated advanced unresectable or metastatic HER2-positive gastric or gastroesophageal junction adenocarcinoma
Enhertu®/fam-trastuzumab deruxtecan-nxki	3rd line (and beyond) Phase 2 (DESTINY-Gastric01)	Antibody-drug conjugates	Daiichi Sankyo	2019	Anti-HER2 antibody (trastuzumab) conjugated to topoisomerase I inhibitor conjugate, deruxtecan, a derivative of exatecan, for treating unresectable or metastatic HER2 positive gastric cancer who received two or more prior anti-HER2-based regimens in the metastatic setting

Table S2. Representative HER2-targeted ADCs and other drugs in clinical trials for treating AGC§

Drug name	Drug Type (Payload)	Identifier/ Phase/Status	Clinical Trial Title	Trial Description/Indication
RC48-ADC	ADC Trastuzimab (MMAE, auristatin E)	NCT03556345 Phase II/III, Completed	A Study of RC48-ADC in Local Advanced/Metastatic Gastric Cancer Subjects With HER2 Overexpression	Evaluate efficacy and safety of a HER2-targeting ADC with a cleavable linker and microtubule inhibitor payload for treating locally advanced or metastatic HER2-positive gastric cancers.
Lenvatinib	Multi-kinase inhibitor + PD-1 Inhibitor	NCT03321630 Phase II, Recruiting	A Study of Lenvatinib, a Multi-targeted Tyrosine Kinase Inhibitor, Combined With Pembrolizumab for Treatment of Metastatic Gastric/Gastro-esophageal Cancer Patients Who Have Progressed on First or Subsequent Line Therapies	Determine PFS, OS, and toxicity rates in advanced gastric/ gastroesophageal patients treated with lenvatinib and pemrolizumab.
GQ1001	ADC Trastuzimab (DM1, maytansinoid mertansine)	NCT04450732 Phase I, Recruiting	Safety of GQ1001 in Adult Patients With HER2+ Advanced Solid Tumors	Phase I dose-finding study in patients with HER2-positive advanced solid tumors.
ARX788	ADC Trastuzimab (MMAF, auristatin F)	NCT03255070 Phase I, Recruiting	Dose-escalation, Expansion Study of ARX788 in Subjects with HER2+ Advanced Solid Tumors	Evaluate RP2D in subjects with advanced HER2 positive cancers and assess anti-cancer activity and safety in HER2-positive tumors.
Vic-trastuzumab duocarmazine (SYD985) + paclitaxel	ADC Trastuzimab (duocarmycin prodrug, seco-duocarmycin-hydroxybenzamide -azaindole)	NCT04602117 Phase I/Ib, recruiting	Evaluating Safety of Weekly Paclitaxel With Trastuzumab Duocarmazine (SYD985) in Patients With Metastatic Cancer: ISPY-P1.01	Assess safety, tolerability, and efficacy of paclitaxel and SYD985 in patients with advanced HER2-positive solid tumors
BPX-603	CAR T cells	NCT04650451 Phase I/II Recruiting	Safety and Activity Study of HER2 Targeted Dual Switch CAR-T Cells (BPX-603) in Subjects with HER2+ Solid Tumors	Assess safety, tolerability, clinical activity of HER2-specific dual-switch CAR T cells and rimiducid in pretreated, locally advanced/ metastatic HER2 amplified or overexpressing tumors
BDC-1001	ADC Trastuzumab (TLR7/8 agonist)	NCT04278144 Phase I/II Recruiting	A First-in-Human Study Using BDC-1001 as a Single Agent and in Combination With Nivolumab in Advanced HER2-Expressing Solid Tumors	Dose escalation study of BDC-1001 as a monotherapy and in combination with nivolumab to determine MTD, RP2D, or MPD for treating patients with selected advanced malignancies.

§*Total of **160** active trials involving various drugs that have been designed to target HER2 receptors expressed on a variety of solid tumor types, but only representative trials focused on advanced gastric cancer (AGC) are tabulated herein. Monoclonal antibodies, antibody-drug conjugates (ADCs), tyrosine kinase inhibitors, chemotherapeutic drugs, CAR T cells, etc., are being used as monotherapies or in combination with other therapeutics to treat HER2-positive solid tumors, including AGC. MTD: maximum tolerated dose; RP2D: recommended Phase 2 dose; MPD: maximum protocol dose; PFS: progression-free survival; OS: overall survival.*

Table S3. In vivo organ-specific time-activity data %ID/g
^{89}Zr-DFO-(Ctr/)-scFv-SG4015-PEG-Cy5-C'dots in NCI-N87 xenografted mice.

^{89}**Zr-DFO-scFv-SG4015-PEG-Cy5-C' dots**												
%ID/g	*3 h*		*24 h*		*48 h*		*72 h*		*144 h*		*192 h*	
	Ave.	*SD*	*Ave.*	*SD*	*Ave.*	*SD*	*Ave.*	*SD*	*Ave.*	*SD*	*Ave.*	*SD*
**Tumor**	6.93	1.37	8.85	1.73	9.97	0.61	10.7	0.59	10.27	0.225	10.8	0.41
**Heart**	16.02	2.01	8.04	1.05	4.82	0.86	3.14	0.51	1.75	0.24	1.15	0.24
**Liver**	5.25	0.45	5.10	0.48	5.00	0.41	4.93	0.40	4.64	0.53	4.65	0.53
**Muscle**	1.34	0.12	1.30	0.11	1.19	0.08	1.13	0.08	1.09	0.05	1.02	0.07

^{89}**Zr-DFO-Ctr/scFv-SG4015-PEG-Cy5-C' dots**												
%ID/g	*3 h*		*24 h*		*48 h*		*72 h*		*144 h*		*192 h*	
	Ave.	*SD*	*Ave.*	*SD*	*Ave.*	*SD*	*Ave.*	*SD*	*Ave.*	*SD*	*Ave.*	*SD*
**Tumor**	4.80	0.85	5.62	0.85	5.77	0.65	5.75	0.49	5.76	0.43	5.14	0.61
**Heart**	15.25	2.26	7.3	1.06	4.48	0.81	2.77	0.49	1.81	0.53	1.25	0.18
**Liver**	5.22	0.28	4.90	0.80	4.69	0.86	4.53	0.75	4.3	0.66	3.94	0.53
**Muscle**	1.28	0.15	1.19	0.13	1.27	0.12	1.1	0.12	1.05	0.05	1.00	0.05

Table S4. ***Ex vivo*** **biodistribution data**
^{89}Zr-DFO-(Ctr)-/scFv-SG4015-PEG-Cy5-C'dots

	**^{89}Zr-DFO-scFv-SG4015-PEG-Cy5-C' dots**		**^{89}Zr-DFO-Ctr/scFv-SG4015-PEG-Cy5-C' dots**	
Organ (%ID/g)	*Mean*	*SD*	*Mean*	*SD*
Tumor	10.78	0.78	5.10	1.30
Blood	0.37	0.16	0.65	0.25
Heart	10.78	0.78	4.05	0.61
Lungs	4.14	0.44	3.49	0.83
Liver	4.61	0.20	4.66	0.14
Spleen	3.76	0.72	3.57	0.52
Stomach	1.09	1.08	1.33	1.04
Sm. Int.	0.91	0.74	1.01	0.36
Lg. Int.	0.87	0.25	1.23	0.49
Kidneys	1.96	1.06	2.65	0.99
Brain	0.06	0.04	0.089	0.023
Feces	0.41	0.45	0.58	0.47
Muscle	1.01	0.62	1.86	1.30
Bone	2.30	0.30	3.23	0.87

Table S5. Radiation dosimetry of ^{89}Zr-DFO-scFv-SG4015-PEG-Cy5-C' dots (DPR=40) in a 70-kg standard man estimated using OLINDA

Tissue	^{89}Zr-DFO-scFv-SG4015-PEG-Cy5-C' dots
	Absorbed Dose
	**Human**
	(rad/mCi)
**Adrenals**	**1.47**
**Brain**	**0.94**
**Gallbladder Wall**	**1.63**
**Colon/Large Intestine**	**1.39**
**Small Intestine**	**1.41**
**Stomach Wall**	**1.33**
**Heart Wall**	**1.35**
**Kidneys**	**1.49**
**Liver**	**1.31**
**Lungs**	**1.85**
**Muscle**	**1.14**
**Pancreas**	**1.46**
**Red Marrow**	**1.11**
**Bone**	**1.21**
**Spleen**	**1.25**
**Thymus**	**1.18**
**Thyroid**	**1.15**
**Urinary Bladder Wall**	**1.33**
**Total Body**	**1.10**
**Effective Dose (rem/mCi)**	**1.00**

n.b. Absorbed Doses in 70-kg Standard Adult Male based on pharmacokinetics in mice and calculated using OLINDA. Mouse absorbed doses were calculated assuming complete local absorption of the particulate radiations only and ignoring the non-self doses.

Table S6. Complete metabolic profiles† of mice injected with saline vehicle (**Control group**, saline vehicle, n=2), scFv-SG4015-PEG-Cy5-C' dots (**Treatment group #1**, 1.2 nmols/mouse, n=2), or scFv-SG4015-PEG-Cy5-C' dots (**Treatment group #2**, 2.5 nmols/mouse, n=2)

	**Treatment**	***Reference***	***#1 Saline***	***#2 Saline***	***#1 scFv-NDC (1.2 nmol)***	***#2 scFv-NDC (1.2 nmol)***	***#1§ scFv-NDC (2.5 nmol)***	***#2 scFv-NDC (2.5 nmol)***
	**Gender**		Female	Female	Female	Female	Female	Female
**Renal**	BUN (mg/dL)	*5.0-28.0*	14.0	20.0	22.0	22.0	---	62.0
	CREA (mg/dL)	*0.2-0.5*	0.22	0.23	0.21	0.26	---	0.25
**Hepatic function**	ALP (U/L)	*105-370*	91.0	107	153	126	102	65.0
	ALT (U/L)	*27-195*	22.0	19.0	32.0	94.0	274	268
	AST (U/L)	*54-77*	52.0	84.0	54.0	206	2087	584
	GGT (U/L)	---	0	0	0	0	---	0
	TBIL (mg/dL)	*0.2-0.6*	0.1	0.1	0.2	0.2	0.3	0.2
	DBIL (mg/dL)	---	0	0	0	0	---	0
	IBIL (mg/dL)	---	0.1	0.1	0.2	0.2	---	0.2
	TP (g/dL)	*4.8-7.2*	5.3	5.2	5.2	5.5	---	5.9
	ALB (g/dL)	*2.4-4.3*	2.8	3.1	3.2	3.4	3.5	3.0
	GLOB (g/dL)	*1.7-2.2*	2.5	2.1	2.0	2.1	---	2.9
	A/G ratio	---	1.1	1.5	1.6	1.6	---	1.0
**Metabolic profile**	P (mg/dL)	*7.3-14.5*	10.0	9.1	8.1	9.6	---	12.7
	Ca (mg/dL)	*9.5-12.5*	10.2	10.2	10.5	11.1	---	11.1
	GLU (mg/dL)	*172-372*	182	161	163	216	---	129
	CHOL (mg/dL)	*55-169*	75.0	66.0	82.0	107	---	191
	TRIG (mg/dL)	*67-289*	149	110	279	258	---	83.0
	CK (U/L)	*428-1609*	45.0	219	50	542	---	446
	TCO2 (mEq/L)	---	22.0	26.0	25.0	20.0	---	13.0
	Na (mEq/L)	*145-181*	157	162	156	156	---	138
	K (mEq/L)	*7.3-11.1*	8.4	6.8	7.6	8.7	---	11.2
	Cl (mEq/L)	*111-134*	112	114	108	111	---	92.0
	Na/K ratio	---	19.0	24.0	21.0	18.0	---	12.0
	Anion Gap	---	31.0	29.0	31.0	34.0	---	44.0

†BUN, blood urea nitrogen; CREA, creatinine; ALP, alkaline phosphatase; ALT, alkaline aminotransferase; AST, aspartate aminotransferase; GGT, gamma-glutamyl transferase; TBIL, total bilirubin; DBIL, direct blirubin; IBIL; indirect bilirubin; TP, total protein; ALB, albumin; GLOB, globulin; A/G, albumin/globulin ratio; P, phosphorus; Ca, calcium, GLU, glucose; CHOL, cholesterol; TRIG, triglycerides; CK, creatine kinase; TCO2, total CO2; Na, sodium; K, potassium; Cl, chloride; Na/K, sodium/potassium ratio

§Limited profile was performed due to low volume of serum available.

Table S7. Complete blood counts of mice injected with saline vehicle (**Control group**, n=2) versus scFv-SG4015-PEG-Cy5-C' dots (**Treatment group #1**, 1.2 nmols/mouse, n=2)

†RBC & Platelet Indices	Reference	*#1 Saline*	*#2 Saline*	*#1 scFv-NDC (1.2 nmol)*	*#2 scFv-NDC (1.2 nmol)*
**RBC (M/uL)**	*6.8-10.5*	9.2	9.5	9.3	9.1
**HGB (g/dL)**	*10.9-15.9*	14.1	14.1	14.7	14.8
**HCT (%)**	*34.9-59.6*	48.1	47.8	50.7	50.2
**MCV (fL)**	*44.5-71.9*	52.0	50.3	54.6	52.2
**MCH (pg)**	*11.5-18.0*	15.2	14.8	15.8	16.3
**MCHC (g/dL)**	*23.0-34.5*	29.3	29.5	29.0	29.5
**RET# (K/μL)**	*294.0-444.0*	609.6	597.2	599.5	729.9
**RET (%)**	*2.6-4.6*	6.6	6.3	6.5	8.0
**PLT (K/uL)**	*376.0-2206.0*	1201.0	872.0	897.0	698.0
**PDW (fL)**	---	10.9	11.6	9.2	13.3

†RBC, red blood cell count; HGB, hemoglobin; HCT, hematocrit; MCV, mean corpuscular volume; MCH, mean corpuscular hemoglobin; MCHC, mean corpuscular hemoglobin concentration; RET#, reticulocyte number per volume; RET, reticulocyte count; PLT, platelet count; PDW, platelet distribution width

Automated Differentials					
**WBC# (K/uL)**	*1.4-10.2*	4.3	4.9	5.2	3.5
**NEUT# (K/uL)**	*0.4-3.6*	1.8	2.3	1.5	1.4
**LYMPH# (K/uL)**	*0.5-6.9*	1.7	1.6	3.1	1.4
**MONO# (K/uL)**	*0.03-0.6*	0.5	0.8	0.3	0.3
**EO# (K/uL)**	*0.01-0.5*	0.2	0.1	0.3	0.3
**BASO# (K/uL)**	*0.0-0.21*	0.0	0.0	0.0	0.2
**NEUT (%)**	*14.0-71.5*	43.2	48.2	29.1	40.2
**LYMPH (%)**	*23.6-79.3*	40.0	33.1	59.4	39.6
**MONO (%)**	*0.9-12.1*	11.0	16.0	6.5	9.5
**EO (%)**	*0.3-7.2*	5.8	2.7	5.0	10.0
**BASO (%)**	*0.0-2.2*	0.0	0.0	0.0	0.6

WBC#, white blood cell count; NEUT#, neutrophil count; LYMPH#, lymphocyte count; MONO#, monocyte count; EO#, eosinophil count; BASO#, basophil count; NEUT (%), neutrophil percentage; LYMPH (%), lymphocyte percentage; MONO (%), monocyte percentage; EO (%), eosinophil percentage; basophil (%), basophil percentage

Table S8. Tumor-specific histologic findings in representative mice injected with saline vehicle (**Control group**, n=2), scFv-SG4015-PEG-Cy5-C‘ dots (**Treatment group #1**, 1.2 nmols/mouse, n=2) or scFv-SG4015-PEG-Cy5-C‘ dots (**Treatment group #2**, 2.5 nmols/mouse, n=2).

Treatment				**Anatomic Pathology**			
	Sex	***Age (weeks)***	***Body Condition Score***	***Body Weight (g)***	***Spleen Weight (g)***	***Gross Finding(s) (tumor)***	***Histologic Findings (tumor)***
Saline vehicle	F	9	3	23.2	NE	Right flank SC tumor (9 x 6 x 3 mm)	AdenoCA, osseous metaplasia of stroma
Saline vehicle	F	9	3	22.7	NE	Right flank SC tumor (10 x 10 x 3 mm)	AdenoCA
scFv-SG4015-PEG-Cy5-C‘ dots (1.2 nmol)	F	11	3	23.1	NE	Right flank SC tumor (3 x 2 x 1 mm)	AdenoCA
scFv-SG4015-PEG-Cy5-C‘ dots (1.2 nmol)	F	11	3	24.3	NE	Right flank SC tumor (5 x 3 x 1 mm)	AdenoCA
scFv-SG4015-PEG-Cy5-C‘ dots (2.5 nmol)	F	11	1	13.5	0.021	Right flank SC tumor (5 x 3 x 2 mm. Small spleen. Scaly trunk skin multifocally	AdenoCA, osseous metaplasia of stroma
scFv-SG4015-PEG-Cy5-C‘ dots (2.5 nmol)	F	11	1	13.3	0.024	Right flank SC tumor 4 x 3 x 1 mm. Small spleen. Scaly trunk skin multifocally	AdenoCA, osseous metaplasia of stroma

SC: subcutaneous, AdenoCA; adenocarcinoma

Table S9. Organ-specific histological findings in mice injected with saline vehicle (**Control group**, n=2), **scFv-SG4015-PEG-Cy5-C' dots** (**Treatment group #1**, 1.2 nmols/mouse, n=2), or **scFv-SG4015-PEG-Cy5-C' dots** (**Treatment group #2**, 2.5 nmols/mouse, n=2).

**Treatment**	**Histologic Finding(s)**					
	#1	***#2***	***#1***	***#2***	***#1***	***#2***
	**scFv-SG4015-PEG-Cy5-C' dots**	**scFv-SG4015-PEG-Cy5-C' dots**	**scFv-SG4015-PEG-Cy5-C' dots**	**scFv-SG4015-PEG-Cy5-C' dots**	**Saline**	**Saline**
Dose	*2.5 nmol*	*2.5 nmol*	*1.2 nmol*	*1.2 nmol*	*--*	*--*
Heart	N	N	NE	NE	NE	NE
Lungs	N	N	NE	NE	NE	NE
Thymus	N	N	NE	NE	NE	NE
Kidneys	N	N	N	N	N	N
Liver	N	N	N	N	N	N
Gallbladder	N	N	N	N	N	N
Stomach	N	N	NE	NE	NE	NE
Duodenum, jejunum, ileum	Marked mucosal degeneration and necrosis, diffuse, characterized by villus atrophy with enterocyte hypetrophy and cytoplasmic vacuolation and eosinophilic inclusions, and crypt necrosis, loss, and hyperplasia.	Marked mucosal degeneration and necrosis, diffuse, characterized by villus atrophy with enterocyte hypetrophy and cytoplasmic vacuolation and eosinophilic inclusions, and crypt necrosis, loss, and hyperplasia.	NE	NE	NE	NE
Cecum	Marked mucosal degeneration and necrosis, diffuse, characterized by superficial mucosal epithelial hypertrophy, and crypt necrosis, loss, and hyperplasia.	Marked mucosal degeneration and necrosis, diffuse, characterized by superficial mucosal epithelial hypertrophy, and crypt necrosis, loss, and hyperplasia.	NE	NE	NE	NE
Colon	Marked mucosal degeneration and necrosis, diffuse, characterized by superficial mucosal epithelial hypertrophy and cytoplasmic vacuolation, and crypt necrosis, loss, and hyperplasia.	Marked mucosal degeneration and necrosis, diffuse, characterized by superficial mucosal epithelial hypertrophy and cytoplasmic vacuolation, and crypt necrosis, loss, and hyperplasia.	NE	NE	NE	NE
LN (mesenteric)	N	N	NE	NE	NE	NE
Salivary glands	N	N	NE	NE	NE	NE
LN (submandibular)	U	N	NE	NE	NE	NE
Uterus	N	N	NE	NE	NE	NE
Cervix	N	N	NE	NE	NE	NE
Vagina	N	N	NE	NE	NE	NE
Bladder	N	N	NE	NE	NE	NE
Spleen	White pulp, lymphoid apoptosis, diffuse, 3.	White pulp, lymphoid apoptosis, diffuse, 3-4.	N	Follicular lymphoid hyperplasia	Follicular lymphoid hyperpla sia	N
Pancreas	N	N	NE	NE	NE	NE
Adrenals	N	N	NE	NE	NE	NE
Ovaries	N	N	NE	NE	NE	NE
Oviducts	N	N	NE	NE	NE	NE
Trachea	N	N	NE	NE	NE	NE
Esophagus	N	N	NE	NE	NE	NE
Thyroid	N	N	NE	NE	NE	NE
Parathyroid	N	N	NE	NE	NE	NE
Skin (trunk)	Acanthosis and hyperkeratosis, D, 3 to 4.	Acanthosis and hyperkeratosis, D, 3.	NE	NE	NE	NE
Mammary glands	N	N	NE	NE	NE	NE

Bones (femur, tibia, sternum, vertebrae)	N	N	NE	NE	NE	NE
Bone marrow (femur, tibia, sternum, vertebrae)	Increased myeloid:erythroid ratio.	N	NE	NE	NE	NE
Stifle joint	N	N	NE	NE	NE	NE
Skeletal muscles (hind limb, spine)	N	N	NE	NE	NE	NE
Nerves (hind limb, spine)	N	N	NE	NE	NE	NE
Spinal Cord	N	N	NE	NE	NE	NE
Oral Cavity	N	N	NE	NE	NE	NE
Teeth	N	N	NE	NE	NE	NE
Nasal Cavity	N	N	NE	NE	NE	NE
Eyes	N	N	NE	NE	NE	NE
Harderian gland	N	N	NE	NE	NE	NE
Bones (skull)	N	N	NE	NE	NE	NE
Pituitary	U	N	NE	NE	NE	NE
Brain	N	N	NE	NE	NE	NE
Ears	N	N	NE	NE	NE	NE

N: Normal; F: Focal; MF: Multifocal; D: Diffuse; UL: Unilateral; BL: Bilateral; 1: Minimal; 2: Mild 3: Moderate; 4: Marked; U: Unavailable; NA: Not applicable; NE: Not evaluated
